# Universal Features of Post-Transcriptional Gene Regulation Are Critical for *Plasmodium* Zygote Development

**DOI:** 10.1371/journal.ppat.1000767

**Published:** 2010-02-12

**Authors:** Gunnar R. Mair, Edwin Lasonder, Lindsey S. Garver, Blandine M. D. Franke-Fayard, Céline K. Carret, Joop C. A. G. Wiegant, Roeland W. Dirks, George Dimopoulos, Chris J. Janse, Andrew P. Waters

**Affiliations:** 1 Leiden Malaria Research Group, Department of Parasitology, Centre for Infectious Diseases, Leiden University Medical Center, Leiden, The Netherlands; 2 Instituto de Medicina Molecular, Unidade de Parasitologia Molecular, Lisboa, Portugal; 3 Centre for Molecular and Biomolecular Informatics, NCMLS, Radboud University Nijmegen Medical Centre, Nijmegen, The Netherlands; 4 W. Harry Feinstone Department of Molecular Microbiology and Immunology, Johns Hopkins Bloomberg School of Public Health, Baltimore, Maryland, United States of America; 5 Department of Molecular Cell Biology, Leiden University Medical Center, Leiden, The Netherlands; 6 Division of Infection and Immunity, Faculty of Biomedical Life Sciences, and Wellcome Centre for Molecular Parasitology, Glasgow Biomedical Research Centre, University of Glasgow, Glasgow, Scotland, United Kingdom; Weill Medical College of Cornell University, United States of America

## Abstract

A universal feature of metazoan sexual development is the generation of oocyte P granules that withhold certain mRNA species from translation to provide coding potential for proteins during early post-fertilization development. Stabilisation of translationally quiescent mRNA pools in female *Plasmodium* gametocytes depends on the RNA helicase DOZI, but the molecular machinery involved in the silencing of transcripts in these protozoans is unknown. Using affinity purification coupled with mass-spectrometric analysis we identify a messenger ribonucleoprotein (mRNP) from *Plasmodium berghei* gametocytes defined by DOZI and the Sm-like factor CITH (homolog of worm CAR-I and fly Trailer Hitch). This mRNP includes 16 major factors, including proteins with homologies to components of metazoan P granules and archaeal proteins. Containing translationally silent transcripts, this mRNP integrates eIF4E and poly(A)-binding protein but excludes P body RNA degradation factors and translation-initiation promoting eIF4G. Gene deletion mutants of 2 core components of this mRNP (DOZI and CITH) are fertilization-competent, but zygotes fail to develop into ookinetes in a female gametocyte-mutant fashion. Through RNA-immunoprecipitation and global expression profiling of CITH-KO mutants we highlight CITH as a crucial repressor of maternally supplied mRNAs. Our data define *Plasmodium* P granules as an ancient mRNP whose protein core has remained evolutionarily conserved from single-cell organisms to germ cells of multi-cellular animals and stores translationally silent mRNAs that are critical for early post-fertilization development during the initial stages of mosquito infection. Therefore, translational repression may offer avenues as a target for the generation of transmission blocking strategies and contribute to limiting the spread of malaria.

## Author Summary

Transmission of malaria relies on ingestion of male and female sexual precursor cells (gametocytes) from the human host by the mosquito vector. Fertilization results in the formation of a diploid zygote that transforms into the ookinete, the motile form of the parasite that is capable of escaping the hostile mosquito midgut environment and truly infecting the mosquito vector. The developmental program of the *Plasmodium* zygote depends on the availability of mRNA pools transcribed and stored, but not translated, in the female gametocyte. Here we identify the core protein factors that co-operate in the assembly of mRNAs into a translationally silent ribonucleoprotein complex. In the absence of either DOZI or CITH—two key molecules within this complex—gametocytes suffer large scale mRNA de-stabilization that does not affect fertilization but culminates in the abortion of ookinete development soon after zygote formation. We characterize large scale, evolutionarily ancient translational silencing as a principal regulatory element during *Plasmodium* sexual development.

## Introduction

Early post-fertilization development in multi-cellular organisms relies on mRNAs supplied in the oocyte in translationally silent P body related storage particles known as P granules. Translation of these maternal mRNA pools depends on fertilization and occurs prior to maternal to zygote transition when transcription from the zygotic genome is initiated [1],[2]. Many P granule components are known [3]–[7] but there is a long-standing question to what constitutes the evolutionarily conserved and essential protein core that controls related events in unicellular eukaryotes during sexual reproduction. In the protozoan *Plasmodium*, formation of a diploid zygote during sexual development coincides with, and is essential for parasite transmission from the human to the mosquito host. *Plasmodium* are haploid throughout most of their life cycle and sexual development in malaria parasites is initiated with the generation of sexual precursor cells, or gametocytes, in the blood of the mammalian host. These mature, haploid male or female forms present distinct proteomic profiles [8] in the absence of sex chromosomes. In the mosquito midgut fertilization yields a diploid zygote that undergoes meiosis without cell division resulting in a tetraploid cell that within 18 hours transforms into the motile ookinete able to truly infect the mosquito. Zygote to ookinete transformation relies on the translational activation of stored, silent mRNAs probably deposited in mRNPs of unknown composition in the female gametocyte [9]. Translationally quiescent mRNAs are found in the cytoplasm of female gametocytes [9]–[12], where long-term maintenance and stabilisation depends on the conserved DEAD-box RNA helicase DOZI [9], a homolog of *Saccharomyces cerevisiae* (yeast) Dhh1p, *Drosophila melanogaster* (fly) Me31b, *Caenorhabditis elegans* (worm) CGH-1 and vertebrate members *Xenopus laevis* P54 and human RCK/P54. In the absence of DOZI, *Plasmodium berghei* zygotes fail to develop into ookinetes, most likely due to a failure to form mRNPs that store and stabilise silenced transcripts. Collectively these destabilized mRNAs encode proteins that are essential for zygote to ookinete transformation during the initial phase of mosquito infection and include adhesins and factors known to be necessary for ookinete motility and traversal through mosquito midgut cells [9]. Translational silencing of certain mRNA species is mediated by a U-rich RNA motif present in the 5′ or 3′ untranslated regions of the implicated mRNAs [13] which also have been shown to specifically silence transgene expression [14].

We provide here the most in-depth characterisation of the protein composition of a P granule to date and demonstrate that the *Plasmodium* particle has a protein core with widespread phylogenetic conservation containing proteins known to form equivalent particles in metazoan oocytes. In addition novel protein components are demonstrated that, although highly conserved, to our knowledge have not been associated with mRNP formation. Functional characterisation of two of the conserved core components revealed distinct phenotypes implying that functionally distinct sub-populations of silenced mRNPs exist.

## Results

### DOZI (CGH-1/Me31b) and CITH (CAR-I/Trailer hitch) define a protozoan, maternal P granule

**Figure 1:**
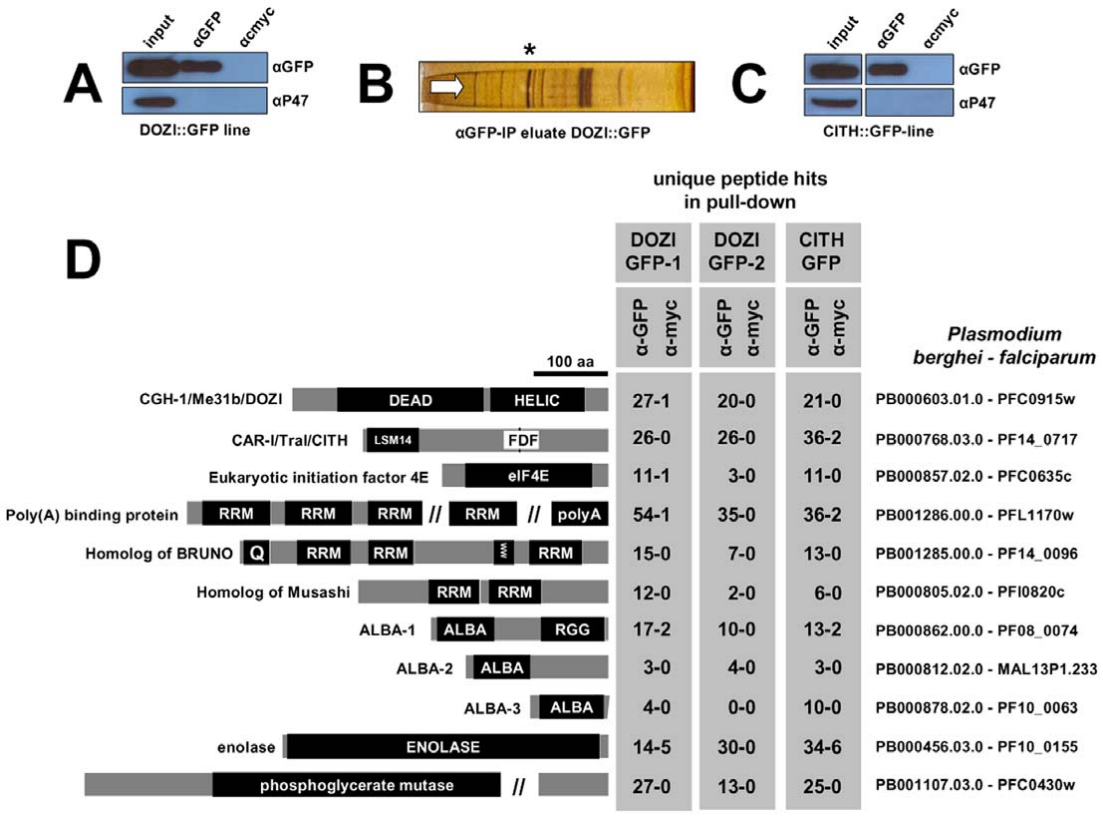
Identification of a multiprotein complex engaged in storage of translationally silent mRNAs in female *Plasmodium* gametocytes. **A and C** Western blot analysis of DOZI and CITH immunoprecipitation (IP) eluates show specific isolation of the respective GFP fusion proteins. P47, a female gametocyte specific protein, did not co-IP and is present only in input fractions. Equivalent amounts were loaded. **B** Silver staining of the DOZI::GFP IP eluate separated on a 12% SDS-PAGE. The asterisk indicates the position/size of the DOZI::GFP fusion protein. **D** Reciprocal IP targeting C-terminal GFP-fusion proteins of DOZI and CITH resulted in the pull down of a set of proteins that were identified using LC-MS/MS. Shown are all proteins with conserved motifs (drawn to scale) and – grey underlaid – the number of unique peptide hits/protein in specific anti-GFP and control IPs. Alignments of these factors and additional *Plasmodium*-specific factors are shown in Table S1, Figures S1, S4, S5, S6, S7, S8, S9 and S10. Homology was defined on amino acid level, as well as domain presence and architecture.

The construction and characterization of a recombinant parasite line that expresses DOZI::GFP from a modified *dozi* allele has been previously reported [9]. Through immunoprecipitation (IP) of DOZI::GFP followed by RNA analysis of IP eluates by Northern and RT-PCR analysis we have previously shown a clear physical association of this DEAD-box RNA helicase with mRNAs known to be translationally silenced in mature, female gametocytes [9]. To define the molecular nature of this putative complex we sought to identify proteins that co-operate with DOZI in the assembly and maintenance of translationally repressed mRNAs. In two independent IP experiments targeting DOZI::GFP a complex from *Plasmodium berghei* gametocytes was purified and analyzed by LC-MS/MS yielding a group of DOZI interaction partners (Figure 1A-B,D; Table S1); one of the co-eluted proteins, PB000768.03.0, showed strong homology with worm CAR-I and fly Trailer Hitch but also *Xenopus* Rap55; these proteins co-localize with their respective DOZI homologues CGH-1 and Me31b to germ cell and P granules [3]–[7] – the *Plasmodium* protein contains both the conserved LSM14 domain and the extended FDF motif (Figures 1D, S1) known to compete with the enhancer of mRNA decapping EDC3 for binding to DDX6 helicases [15], and is therefore designated CITH (CAR-I/Trailer Hitch Homolog; Figure S1). To corroborate the DOZI pull down results, a reciprocal IP (Figure 1C) was performed using lysates from gametocytes of a transgenic *P. berghei* line expressing only C-terminally GFP-tagged CITH (Figure S2). Mass-spectrometric analysis of the CITH::GFP pull down resulted in the identification of the same 16 core factors (Figures 1D and Table S1). A linear regression analysis revealed no bias towards high molecular weight or abundant proteins (Figure S3); 7 of the 16 proteins were previously found to be sexual stage specific in *P. berghei* (PB000695.03.0, PB000120.01.0, PB001107.03.0, PB000768.03.0, PB000603.01.0, PB000647.02.0, PB000124.01.0) [8].

**Figure 2:**
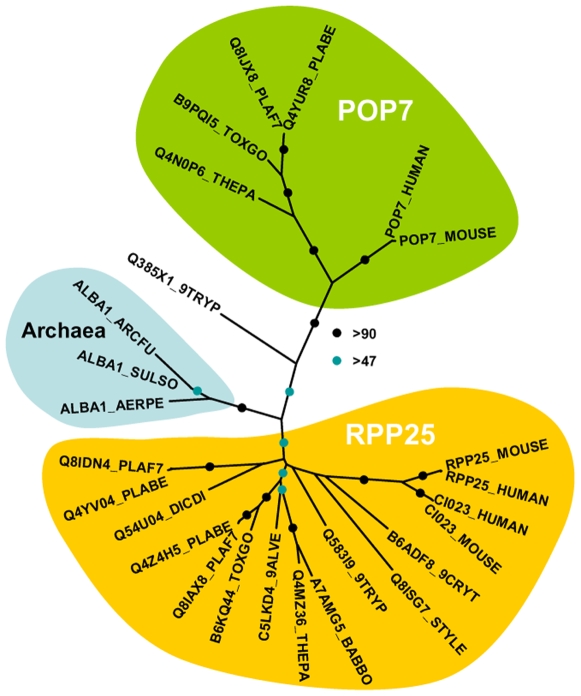
Phylogenetic position of *Plasmodium* Alba-domain proteins. We generated a multiple sequence alignment of the conserved region of a range of Alba-domain proteins (PFAM PF01918). The phylogenetic tree was generated with PHYML. Bootstrap values (100 replicates) are based on neighbor joining and maximum likelihood analyses. Accession numbers are from uniprot; species abbreviations are for *Perkinsus marinus* (9ALVE), *Cryptosporidium muris* (9CRYT), *Trypanosoma brucei* (9TRYP), *Aeropyrum pernix* (AERPE), *Archaeoglobus fulgidus* (ARCFU), *Babesia bovis* (BABBO), *Dictyostelium discoideum* (DICDI), *Plasmodium berghei* (PLABE), *Plasmodium falciparum* (PLAF7), *Stylonychia lemnae* (STYLE), *Sulfolobus solfataricus* (SULSO), *Theileria parva* (THEPA) and *Toxoplasma gondii* (TOXGO).

The analysis of the DOZI and CITH pull down eluates gives an unprecedented depth of characterisation of the protein component of a P granule (Figure 1D). Among the DOZI and CITH-associated proteins identified with a high level of confidence are the *Plasmodium* homologs of the 5′ cap binding protein eIF4E (PB000857.02.0, Figure S4) and poly(A) binding protein (PABP; PB001286.00.0, Figure S5). Both are commonly found in mammalian stress granules [16] and PABP protects mRNAs from de-adenylation and degradation. In addition we identified orthologs of proteins that function as translational regulators in metazoans; one protein with strong homology to the ELAV/BRUNO-family and a second with weak homology to Musashi: the *Plasmodium* proteins are Homolog of *Drosophila* BRUNO (HoBo, PB001285.00.0, Figure S6), and Homolog of Musashi with two RNA recognition motifs (HoMu, PB000805.02.0 Figure S7). *Drosophila* BRUNO targets mRNAs such as *oskar* containing the 3′ UTR BRUNO response element for silencing [17], while Musashi is a translational regulator found to compete with eIF4G for PABP-binding in neural stem cells [18]. For the first time we identify in maternal mRNPs Alba domain proteins (Acetylation Lowers Binding Affinity); the entire complement of *P. berghei* Alba domain proteins (Alba-1, PB000862.00.0; Alba-2, PB000812.02.0 and Alba-3, PB000878.02.0) (Figure 1D) co-IPs with DOZI and CITH. These proteins are small, with predicted molecular weights of 27, 23 and 12 kDa, respectively with a single, N-terminal Alba domain (Figure S8). Alba-1 contains multiple RGG-box RNA binding domains at the C-terminus, a characteristic of plant and protozoan proteins. Phylogenetic analyses places Alba-1 and Alba-2 into the MDP2/Rpp25 superfamily, whereas Alba-3 belongs to the POP7/Rpp20 group (Figures 2 and S8) [19]. Interestingly, within the Apicomplexa only the genus *Plasmodium* appears to have 2 members within the MDP2 group. Two enzymes potentially associated with glycolysis were identified, i.e. a member of the phosphoglycerate mutase (PGAM) family (PB001107.03.0, Figure S9) and enolase (PB000456.03.0, Figure S10). Lastly, 5 abundant proteins show no or little homology to proteins outside *Plasmodium* spp.; they are PB000695.03.0, PB000120.01.0, PB000647.02.0, PB000124.01.0 and PB000642.01.0.

### DOZI and CITH complexes contain translationally repressed mRNAs

**Figure 3:**
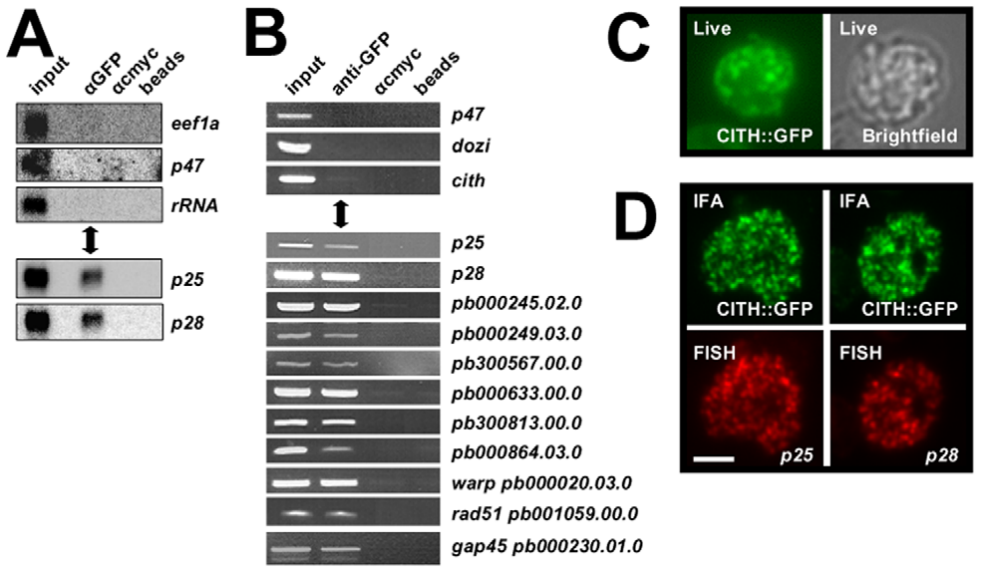
CITH co-localizes with translationally repressed mRNAs. **A** Northern analysis of CITH::GFP IP eluates show specific co-elution of translationally repressed mRNAs *p25* and *p28*, but not transcripts known to be translated (*p47*, *eef1a*) or ribosomal RNA. Equivalent amounts of eluates and input were loaded. **B** RT-PCR analyses show specific enrichment of transcripts known to be translationally repressed in the anti-GFP pull down; transcripts of expressed proteins (P47, DOZI, CITH) are absent. **C** The CITH::GFP fusion protein is present in characteristic foci in the cytoplasm of live parasites. **D** Fluorescent *in situ* hybridization combined with immunofluorescence analysis shows overlapping signals for *p25* and *p28* mRNAs and CITH::GFP. Scale bar  = 4 µm.

Consistent with the similarities in protein content of the DOZI and CITH IPs, the same silenced mRNA species associated with DOZI [9] were also found to co-elute with CITH by Northern analysis and RT-PCR (Figures 3A and B) but not transcripts known to be translated in gametocytes. The RNA-IP experiments indicate that CITH together with DOZI resides in a stable, translationally quiescent P body-like structure. The bulk of DOZI and CITH protein is present in female gametocytes as shown by immunofluorescence and proteome analysis of asexual stage and purified gametocytes [8] and expression of both proteins persists throughout ookinete development [8],[13]. The *P. falciparum* DOZI ortholog (PFC0915w) has also been detected in sporozoites [20]. We consistently observe large CITH::GFP granules in live gametocyte preparations (Figure 3C); in addition the protein overlaps partially with two of the best characterised maternally silenced mRNAs, *p25* and *p28*, in cytoplasmic foci with a speckled appearance typical for such mRNPs (Figure 3D).

### DOZI and CITH gene deletion mutants are fertile but abort zygote to ookinete transformation

**Figure 4:**
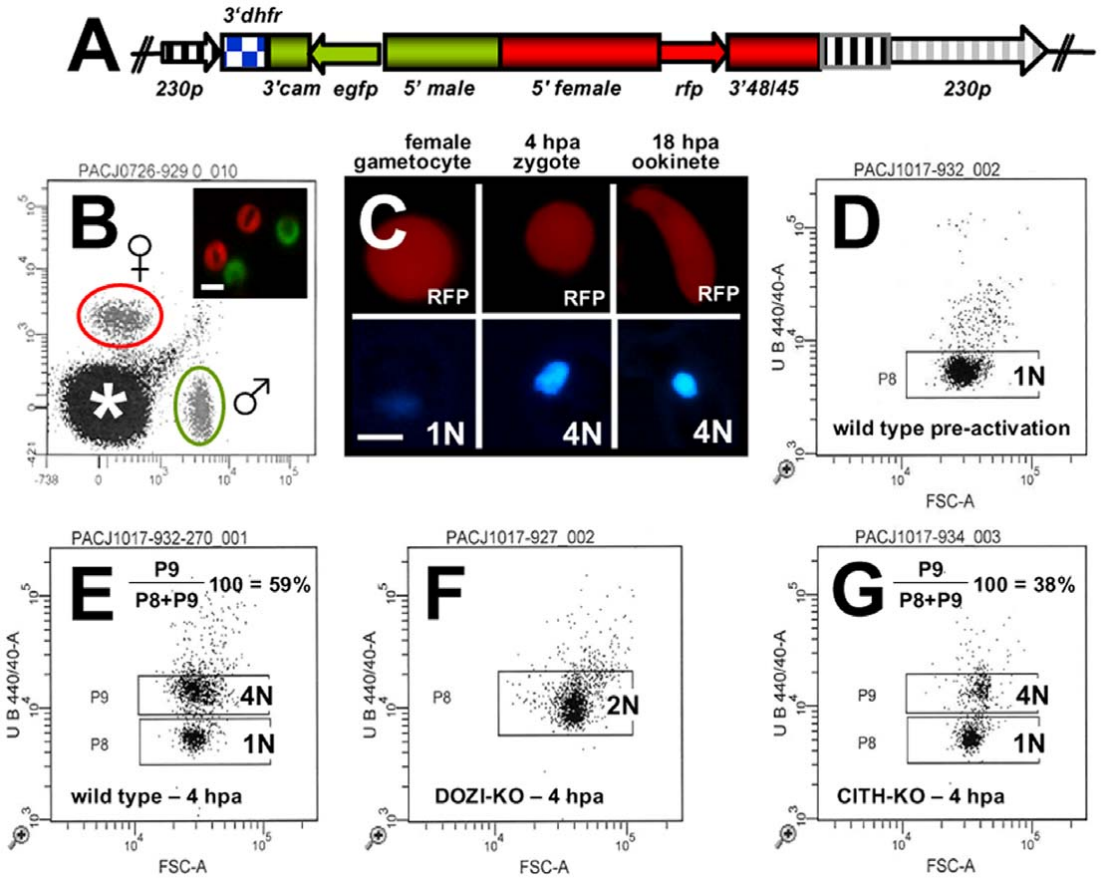
Zygotes formed by Δ*pbcith* and Δ*pbdozi* gene deletion mutants abort zygote to ookinete transformation. **A** Schematic representation of the vector used to introduce the *gfp/rfp* male/female expression cassette into the *p230p* locus. **B** Identification of populations of RFP+ female gametocytes and GFP+ males by FACS in blood infected with parasites of line 820cl1m1cl1. Gametocyte populations are clearly separated from the population of red blood cells and red blood cells infected with the asexual bloods stages (asterisk). The inset shows male (GFP+) and female gametocytes (RFP+). Scale bar  = 4 µm. **C** RFP and Hoechst 33258 staining of female gametocyte, zygote and ookinete of line 820cl1m1cl. Zygotes and ookinetes were collected at 4 hours post activation (hpa) and 18 hpa, respectively. Note the increased Hoechst fluorescence intensity of the nuclei of zygotes and ookinetes as a result of fertilization and meiotic DNA replication, resulting in tetraploid nuclei. Scale bar  = 2 µm. **D and E** FACS analysis of Hoechst fluorescence intensity of wild type female gametes (D) and zygotes (E). In (D) haploid female gametes are shown before fertilization and (E) shows unfertilized females (1N) and tetraploid zygotes (4N) collected at 4 hpa. **F** Δ*pbdozi* females are fertilized as shown by the doubling in DNA content, but do not achieve tetraploidy. **G** Females of Δ*pbcith* mutants develop into tetraploid forms.

The similarities of mRNA and major protein contents of DOZI and CITH IPs indicate that they are largely a component of the same mRNP responsible for post-transcriptional regulation of gene expression at the level of translation initially defined by DOZI. As zygotes lacking DOZI fail to progress through meiosis and are unable to transform into ookinetes [9] we wanted to identify any possible effects on zygote to ookinete transformation in the absence of CITH. Mutant parasite lines that lack *pbcith* (Δ*pbcith*) (Figure S11) showed normal asexual blood stage development and wild type production of gametocytes and gametes but failed to generate ookinetes (data not shown). To analyse in greater detail possible fertilization and meiosis defects we generated *Δpbcith* and *Δpbdozi* lines [934cl1(Figure S12) and line 927cl1 (Figure S13)], respectively] in a reporter line with red fluorescent protein (RFP) expression exclusive to female gametocytes, that persists throughout ookinete development (Figures 4A-C and S14; see also Protocol S1 and www.pberghei.eu). In addition to RFP under the control of the female-specific promoter of gene *pb000504.02.0*, GFP is driven by the male-specific promoter of gene *pb000791.03.0*. Both transgenes are stably introduced into the *230p* locus on chromosome 2. Therefore, stage specific RFP expression permits identification of female gametes and zygotes after fertilization for FACS-analysis of their DNA contents by Hoechst staining. Such analyses made 4 hours after activation, when the zygote normally has completed meiosis, are able to reveal cell ploidy and are therefore a quantitative indicator of fertilization success and zygote development from the diploid to the tetraploid state (Figures 4D and E). These studies confirmed that the Δ*pbdozi* line fertilises normally when compared to wild type; the male and female nuclei fuse but fail to complete meiotic replication and remain diploid (Figure 4F). Surprisingly, Δ*pbcith* mutants present a different phenotype where they also fertilise normally yet progress through meiotic DNA replication to establish tetraploidy (Figure 4G). However, further development of the spherical zygote into the motile, banana-shaped ookinete is aborted soon after zygote stage I/II, before gross morphological changes become apparent [21]. Consequently, neither gene deletion mutants are able to transform into ookinetes (Table 1A). Standard cross-fertilization assays [8] in which gametes of Δ*pbcith* were crossed with either fertile male (parasite line 137.1, Δ*p47*) or female gametes (parasite line 370.1, Δ*p48/45*) demonstrated that male gametes are unaffected by the absence of CITH – the block in development of the zygote is due to the absence of the protein provided by female gametes resulting in sterility (Table 1B). Therefore, despite the clear similarities in proteins associated with DOZI and CITH, their maternal origin and essential role in zygote to ookinete transformation, the specific effects on early zygote development are different.

**Table 1:** Ookinete formation in wild type and mutant parasite lines.

A	Ookinete conversion rates (%)	B	Ookinete conversion rates (%)	
			Fertile male 137.1	Fertile female 370.1
wild type	37±5			
927.1 DOZI-KO	none	927.1 DOZI-KO	none	34±2
934.1 CITH-KO	none	934.1 CITH-KO	none	34±10

**A** Neither Δ*pbdozi* nor Δ*pbcith* mutants transform into ookinetes. **B** Cross-fertilization of mutant with wild type gametes indicates that female Δ*pbcith* and Δ*pbdozi* mutants are deficient in ookinete development, whereas male gametes from either line are fertile. Conversion rates are mean counted as a percentage of initial female gametocytes ± s.d.

### Maternal lethal effects of CITH and DOZI

**Figure 5:**
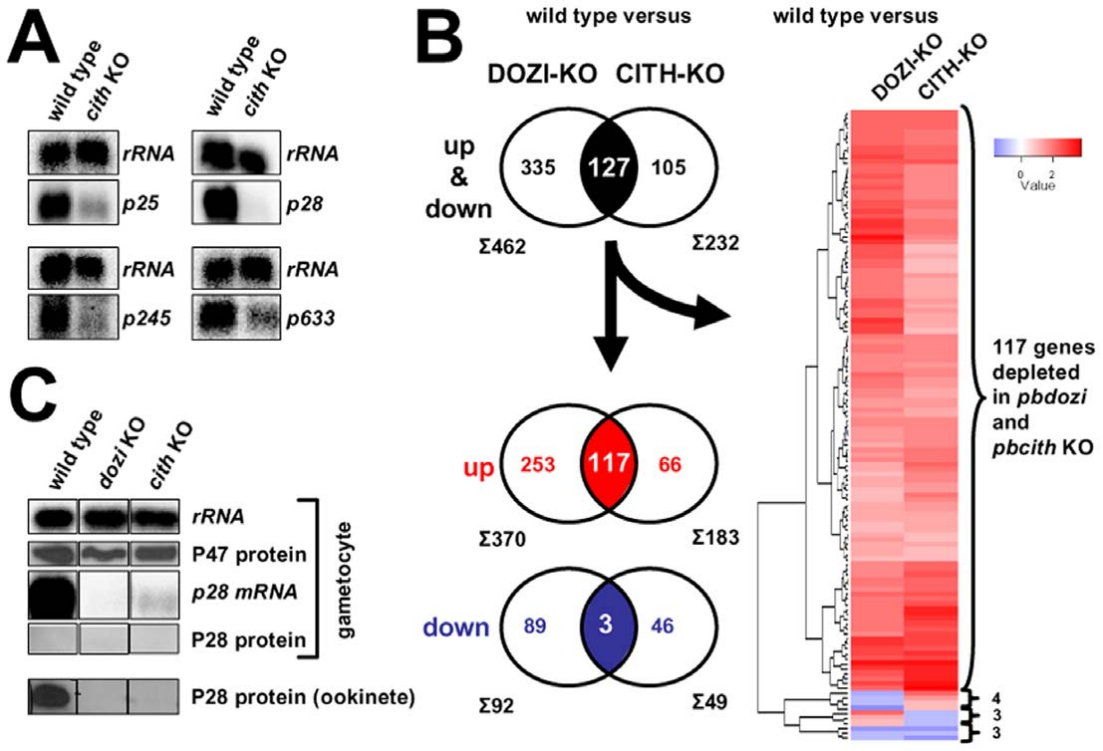
CITH and DOZI gene deletion mutant gametocytes suffer substantial mRNA loss. **A** Northern blot analysis of four translationally repressed transcripts in wild type and Δ*pbcith* mutants shows a clear de-stabilizing effect in the absence of CITH in gametocytes. **B** Venn diagrams of all 2-fold differentially expressed genes common to DOZI and CITH KO parasites. Hierarchical cluster analysis of the differentially expressed genes common to both lists. **C** Absence of DOZI or CITH does not result in the precocious translation of *p28* mRNA into protein in female gametocytes.

In Δ*pbdozi* gametocytes the expression levels of 370 transcripts (6% of all *Plasmodium* genes) were more than 2-fold reduced when compared to wild type gametocytes [9]. In order to identify if similar molecular effects contribute to the observed developmental defect in the *pbcith* mutant parasite, we performed a small Northern survey of abundant but translationally repressed mRNAs, among them the hallmark gene *p28*. Using RNA isolated from gametocytes, *p28* together with 3 additional transcripts appeared less abundant in the CITH KO parasites, indicating a destabilising effect on these mRNAs in the absence of CITH (Figure 5A), thus prompting us to perform a global transcriptome profiling of gametocyte RNA and identify whether mRNA destabilisation is a global phenomenon. Microarray hybridisation of Δ*pbcith* mutants revealed that the expression levels of 232 transcripts were significantly changed, with 183 mRNAs more than 2-fold down regulated (DR) representing 50% of the Δ*pbdozi* number (Table S2). As in Δ*pbdozi*, several transcripts (46) were unexpectedly up-regulated (UR) in the absence of CITH. In total, 82% of the protein products of all differentially expressed transcripts are absent from the gametocyte proteome [8] indicating that these transcripts are stabilised and silenced in a CITH dependent manner. 127 mRNAs were common to the DOZI and CITH data sets (Figure 5B and Table S3) although neither the degree of a given individual transcript nor the rank order was consistent between the two mutants (R^2^ = 0.25; Pearson r = 0.50, Figure S15A). 117 are DR in both KOs, 3 were UR, whereas 7 transcripts are inversely modulated. Gene Ontology (GO) enrichment analysis (Figure S15B) revealed no bias most likely due to incomplete and therefore high number of hypothetical annotations (89 genes). However, 21 proteins are predicted to contain a signal peptide and 24 contain one or more trans-membrane domains suggesting cell surface localisation; among those are known adhesins – factors that function in host-cell receptor interactions and promote successful invasion of the midgut epithelium resulting in infection of the mosquito – and include *p25*, *p28*, *warp*, *p36,* and members of the *pb-fam-5/cpw-wpc* and *lap* families including *ccp2* and *lap5*. In addition 5 alveolins (membrane sac proteins), inner membrane complex 1b protein, gliding motility associated protein *gap45*, 3 protein kinases, a member of the *ap2/erf* family of transcription factors (*api-o*) that initiates transcription of ookinete-specific genes [22] and *rad51* are DR; finally, so are the 9 mRNAs previously shown to share a cis-acting RNA motif that confers silencing in female gametocytes [13],[14]. In total, only 8% of the common differentially expressed genes are present in the *Plasmodium* gametocyte proteome [8] suggesting that CITH and DOZI co-operate in the protection from degradation of translationally quiescent, maternally supplied mRNAs.

### Are there DOZI and CITH-specific mRNPs?

While a large number of genes are co-regulated by DOZI and CITH, the differences in the repertoire of DR mRNAs between Δ*pbdozi* and Δ*pbcith* gametocytes may indicate the presence of mRNPs with distinct mRNA content that is reflected in the observed developmental defects. A number of meiosis-associated transcripts were exclusively depleted in the Δ*pbdozi* mutant which arrests before completion of meiosis (Table S4); these include the RNA-binding protein *mei2* (*pb001281.02.0*) and the chromosome segregation myosin-ATPase (*pb300220.00.0*). Three additional AP2/ERF transcription factors (*pb00974.00.0*, *pb001077.01.0*, *pb300561.00.0*) [23],[24] as well as 6 mRNAs encoding Zn-finger domain proteins are significantly destabilized, and their protein products are likely to play a role in the activation of the zygotic genome.

### Failure to establish the P granule results in *p28* destabilization but not translation

*p28* is one of the first and best characterized translationally repressed mRNAs. Its protein product, which is displayed on the surface of the ookinete, plays an important role during mosquito midgut invasion making it a promising candidate for transmission blocking intervention [25]. DOZI and CITH gene deletion mutants fail to stabilize *p28* and this failure could potentially lead to the precocious translation of P28 protein in blood stage gametocytes. Therefore we wanted to know the fate of *p28* mRNA in DOZI and CITH gene deletion mutants. As shown in Figure 5C, absence of either factor does not result in P28 protein translation indicating that the mRNA is most likely degraded when not stored, and unable to resume translation.

## Discussion

Zygote to ookinete transformation occurs over an 18-hour period in the female mosquito midgut which is a hostile environment actively engaged in the systematic destruction of cellular material in order to provide nutrition for mosquito egg production and maturation. It is known that animal oocytes store mRNA in order to bypass the need for transcription during early embryogenesis before activation of the newly formed zygotic genome [1],[2],[26]; whilst this requirement holds true for the parasite, *Plasmodium* zygotes undergo meiosis within 4 hours of fertilization, which is followed by the timely formation of the ookinete – a motile parasite form able to actively escape the hostile mosquito midgut environment by penetrating the surrounding epithelium; these different developmental requirements may influence the composition of the P granule.

### Maternal mRNA storage depends on a protein core conserved from unicellular organisms to germ cells of metazoans

The LC-MS/MS analysis of the DOZI and CITH-associated proteins revealed 16 common, major protein factors. They could be grouped into a number of different classes based on predicted activity: 1. Proteins with homology to constituents of metazoan P granules; these proteins (DOZI, CITH, eIF4E, HoBo/BRUNO, and HoMu/Musashi) have been demonstrated to be present in mRNPs from various organisms although never in a single mRNP as presented in this study. The presence of PABP in *Plasmodium* P granules and metazoan germ cell granules [4],[7] may indicate an intrinsic readiness of the particle to present repressed transcripts to the ribosome in response to the identified need for speed at the same time protecting mRNAs from degradation. 2. Alba domain containing proteins have not been identified in association with mRNPs before. In Archaea they are known to bind RNA, principally ribosomal RNA [27], and eukaryotic POP7 and Rpp25-related proteins take part in tRNA and rRNA processing, while ciliate MDP2 is a factor in macronuclear development [28],[29]. In the Archaea Alba regulates transcription through chromatin organisation where DNA binding affinity is controlled by the sirtuin SIR2 and Pat [30],[31]. In *Plasmodium* SIR2 regulates the expression of sub-telomerically located genes of multigene families encoding variant antigens [32]–[37]. However, the association of Alba proteins with factors that regulate translational repression (TR) might indicate that sirtuin de-acetylases and their counterpart acetylases also have a post-transcriptional role in the control of gene expression in *Plasmodium.* 3. Two proteins associated with glycolysis were identified independently in DOZI and CITH IP-eluates, enolase and a member of the PGAM family. The role in TR implied by their association with the DOZI/CITH mRNP found in *Plasmodium* and possible moonlighting functions are currently obscure and requires further attention. However, it is well established that enolase has roles in biological processes besides its role in glycolysis, including transcription, heat shock, autoimmunity and it may also serve as a plasminogen receptor and function in the bacterial degradasome [38]. PGAM family members are known to participate in complexes including those which repress gene expression at the level of transcription [39].

### P body RNA degradation factors are absent from *Plasmodium* maternal P granules

CGH-1/Me31b/Dhh1 are present in diverse, functionally distinct P body families; these include maternal P [5]–[7] and stress granules [16], but also co-localisation with RNA de-capping factors such as DCP1/2 [40] and presence in a miRNA-induced silencing complex [41] has been shown. It is significant that in this study and in contrast to the identification of proteins that are present in P bodies and stress granules, no factors were identified that constitute the core of P bodies during RNA degradation [42] and that interact with DOZI. *Plasmodium* homologs of most proteins with exonuclease activity and involved in mRNA de-adenylation and decapping (e.g. XRN1, Lsm1-7, DCP1 and 2, UPF1-3) are readily identified in the annotated genome (Table S5). Yet they were absent from the IP eluates, confirming that gametocyte mRNPs defined by DOZI and CITH contain stable, translationally repressed transcripts awaiting re-activation and translation following fertilization. This emphasizes that in the context of the gametocyte the activity of DOZI is predominantly one of mRNA storage and not degradation. It is intriguing that specific and overlapping, but also non-identical mRNA populations are destabilized in the gametocyte in the absence of DOZI and/or CITH. Our data support the existence of different forms of P granules that are defined by the destabilized mRNA populations in the CITH and DOZI depleted mutants and the observed different developmental defects of these mutants.

### Repression acts on diverse mRNA populations

Our experiments detailing the destabilising effect on a substantial mRNA population of the gametocyte show that silencing influences diverse processes during zygote to ookinete formation. For example, the newly formed zygote is provided with coding potential for proteins known and likely to be involved in ookinete development, for instance in the activation of the zygotic genome; the presence of AP2/ERF transcription factors (TF) and DNA Zinc-finger binding domains in the down regulated set of genes indicate that these factors are already supplied in the female gametocyte. One of these TF (API-O) promotes transcription of genes during ookinete development and is present in DOZI-defined mRNPs [22]. Secondly, 25% of the commonly down regulated mRNAs encode proteins with known and predicted surface localisation. In the case of P25 and P28 it is well established that they facilitate the escape of the parasite from the hostile mosquito midgut milieu [43]; transcription of these mRNAs in blood stage gametocytes and subsequent retention in silent mRNPs provides rapid access of these transcripts to ribosomes and therefore the production of these essential proteins. However, storage in P granules may also contribute to immune evasion mechanisms in the mammalian host. Antibodies to P25 and P28 are promising transmission blocking vaccines – their presence in a mosquito blood meal substantially reduces the ability of the parasite to infect the mosquito vector [44],[45]. We have shown here and previously [9] that prevention of complex formation in gametocytes in CITH and DOZI KO mutants induces degradation and not translation of *p25* and *p28* mRNAs, while female gametocytes are fully translation competent as shown in numerous GFP transgene experiments [8],[46],[47]. In addition *gfp* translation can be abrogated when tethered to the 3′ UTR of *p28* [46]. It is therefore tempting to speculate that the parasite has evolved a fail-safe mechanism that results in the degradation of mRNAs meant to be silenced in case the transcript fails to be stored in P granules.

### The maternal P granule is evolutionarily ancient

Our experiments corroborate that protozoans, like female germ cells of higher eukaryotes, rely on the storage of mRNA in the female gamete during sexual reproduction, specifically during early post-fertilization development. DOZI and CITH in *Plasmodium* are *bona fide* translational repressors that contribute to successful ookinete development in and infection of the mosquito vector by storing a substantial mRNA population in pre-fertilization, female gametocytes. In worm oocytes CGH-1 granules associate with roughly 6% of all known expressed genes [4] compared with approximately 7% down regulated mRNAs in *Plasmodium* gametocytes. Although the protected mRNA species are not conserved in the 2 organisms, the fundamental DOZI/CGH-1-dependent protection of transcripts is. The normal generation of ookinetes from crossings of CITH and DOZI-KO male gametes wild type female gametes also show that the observed sterile developmental phenotype is entirely a maternal effect previously identified for *Drosophila* Trailer hitch where mutant female flies are sterile and present defects in egg laying [7],[48].

### Conclusions

Translational repression (TR) is an important mechanism of post-transcriptional gene regulation that in metazoan germ-line but also somatic cells generates spatial and temporal protein diversity that is independent from transcriptional control and protein targeting signals. Our data demonstrate that such mRNPs in the protozoan *Plasmodium* rely on an evolutionarily conserved and ancient protein core that secures mRNP integrity and future translatability of stored mRNAs in a DOZI and CITH-dependent manner. The relatively tractable nature of the *P. berghei* malaria model will allow a detailed dissection of the role of conserved and species-specific proteins in TR. Furthermore, the novel involvement of Alba proteins in TR and the coupling of post-transcriptional modifications to signalling as an effector of TR may yet prove to be informative of control of TR in general.

## Materials and Methods

### Ethics statement for animal experimentation

All studies in which animals are involved were performed according to the regulations of the Dutch “Experiments on Animals Act” and European regulations (EU directive no. 86/609 regarding the Protection of Animals used for Experimental and Other Scientific Purposes) and approved by the Animal Experiments Committee of the LUMC (ADEC; established under section 18 of the “Experiments on Animals Act” and registered at the Dutch Inspectorate for Health Protection and Veterinary Public Health, which is part of the Ministry of Health, Welfare and Sport).

### Generation of *Plasmodium berghei* mutants expressing C-terminally GFP-tagged CITH and DOZI

The mutant parasite line that expresses a C-terminally GFP-tagged version of DOZI (683cl4) has been described [9]. CITH::GFP parasites [line 909cl1 (Figure S2)] were generated in the parent reference line of the ANKA strain cl15cy1 with a GFP-tagging vector pL1200 containing a single genomic targeting region for single cross-over homologous recombination generated by PCR with primers 2831-*Eco*RI and 2832-*Not*I. Mutant parasites express only the GFP-tagged gene. Targeting regions, primers used and genotype analysis are shown in Table S6. Please also refer to www.pberghei.eu and Table S7 for mutant *P. berghei* parasites lines used in this study.

### Immunoprecipitation (IP) experiments and mass-spectrometric analysis

IP of DOZI::GFP and CITH::GFP complexes was performed on whole cell lysates from purified gametocytes as described in Supplementary Online Material of reference [9] using monoclonal anti-GFP antibodies (Roche) and control anti-cmyc antibodies (SIGMA). Processing of eluates and mass-spectrometric analysis by LTQ-FT are described in Protocol S1. Total RNA from IP eluates was extracted with TRIzol and used in Northern blot analysis and RT-PCR. Primers used are shown in Table S6. Western blot analysis of IP eluates was performed using monoclonal anti-GFP antibodies (Roche) and anti-P47 [49] as described [9].

### Generation of mutants deficient in expressing CITH and DOZI

*pbcith* (*pb000768.03.0*) was targeted for genetic disruption by standard double-crossover homologous recombination with vectors containing the *Toxoplasma gondii* (tg) dhfr/ts selection cassette flanked by targeting sequences of the corresponding ORF (Figure S11). Targeting regions were generated by PCR with primers 2773-*Asp*718I and 2774-*Hind*III, and 2775-*Eco*RI and 2776-*Not*I. Transfection and selection of mutant parasites was performed using genetic modification technology developed for *P. berghei* [50]. Correct integration of plasmids and disruption of the genes was verified by Southern analysis of separated chromosomes and diagnostic PCRs, and Northern analysis. Targeting regions, primers used and gels are shown in Figure S12. *pbcith* was disrupted in three independent experiments (856, 893, 934); lines 856 and 893 were generated in a wild type reference line of the ANKA strain (507cl1) that constitutively expresses a *gfp* transgene under the control of the *eef1a* promoter, stably integrated into the *pb230p* locus without use of a drug-selectable marker [50]. Line 934 was generated in a second wild type reference line (line 820cl1m1cl1; Figure S12) which contains *gfp* and *rfp* transgenes under the control of male (*pb000791.03.0*) and female (*pb000504.02.0*) specific promoters, respectively, stably integrated into the *pb230p* locus without the use of a drug selectable marker (see Protocol S1 *Generation of a reporter P. berghei line that expresses RFP in female gametocytes, gametes and zygotes* for further details of the generation of this line). Cloned parasite lines of transfection 856 and 934 were obtained by limiting dilution and used for further analysis of the phenotype (Figure S12). Parasite lines in which *pbdozi* has been disrupted in the ANKA strain have been described. In addition, we disrupted for this study *pbdozi* in the reference line 820cl1m1cl1 (927cl1; Figure S13) using the same DNA construct as described [9].

### Generation of a reporter *P. berghei* line (820cl1m1cl1) that expresses RFP in female gametocytes, gametes and zygotes

Generation of a reporter *P. berghei* line (820cl1m1cl1) is described in detail in Protocol S1 and Figure S14.

### *In vitro* (cross) fertilization and ookinete maturation assays

The fertility of wild type and mutant gamete populations was analysed by standard *in vitro* fertilization and ookinete maturation assays [8],[49] from highly pure gametocyte populations [51]. Fertility (ookinete conversion) of gametes is defined as the percentage of female gametes that develop into mature ookinetes determined by counting female gametes and mature ookinetes in Giemsa stained blood smears 16–18 hours after gametocyte activation. Fertility of individual sexes (macro- and micro-gametes) was determined by *in vitro* cross-fertilization studies in which gametes are cross-fertilised with gametes of lines that produce only fertile male (270cl1) or only fertile female gametes (137cl1) [8],[9],[49]. All assays were done in triplicate on multiple occasions in independent experiments.

### Analysis of fertilization and meiosis by FACS

Fertilization and meiosis in wild type and mutant lines was inferred from their DNA content (or ploidy) determined by FACS measurement of fluorescence intensity of cells stained with the DNA-specific dye Hoechst-33258. For these experiments we used the mutant lines generated in the parent line 820cl1m1cl (see Protocol S1) that expresses RFP in the female gametocyte/gamete and continues into the zygote and ookinete. Stage specific RFP expression allows selection of female gametes and zygote stages in the process of FACS-analysis of the DNA content of cells. Activation of gametocytes was performed in *in vitro* (cross) fertilization and ookinete maturation assays as described above. At 4 hours post activation (hpa) cells were stained for one hour at room temperature with Hoechst-33258 (10 µM) and analysed at room temperature by FACS using a LSR-II flow cytometer (Becton Dickinson) with the following filters and settings: UB 440/40 (Hoechst)|400 (parameter)|5000 (threshold); BE 575/26 (RFP)|500|5000; BF 530/30 (GFP)|500|5000; FSC|250|2000; SSC|200|5000. Cells for Hoechst analysis were selected on size by gating on FSC and SSC. Per sample 10.000–500.000 cells were analyzed (medium flow speed, sample pressure: medium) and all measurements were performed on triplicate cultures. Female cells were selected for Hoechst-33258 fluorescence intensity based on their RFP expression (Figure 4). To determine the Hoechst-fluorescence intensity from the populations of unfertilized female gametes and zygotes gates were set as shown in the Figures. Data processing and analysis was performed using the program FlowJo (www.flowjo.com).

### Microarray analysis of CITH KO parasite lines

Hybridisation with total RNA from wild type and CITH KO mutants were done in biological triplicates on glass slides from Agilent Technologies (www.agilent.com) containing sixty-mer oligonucleotides for the 5283 predicted *P. berghei* transcripts as described in Supplementary Online Material of reference [9]. Transcripts were tested for differential abundance through competitive hybridization of WT vs. CITH-KO labeled RNAs. Significance of expression was determined using TIGR MIDAS and MeV software and a LOWESS normalization method (p value <.05). Genes found differentially expressed in both wild type vs. DOZI-KO and wild type vs. CITH-KO and with a fold change cut-off of 2, were clustered using a Euclidean distance matrix of log2 ratio of genes for each condition. The heat map was drawn using the gplots package of R/Bioconductor [52] with up-regulated genes in the wild type parasites in red and down regulated genes in the wild type parasites in blue.

### Oligonucleotide primers

For primers used in the generation of plasmid vectors, templates for probes for Northern and Southern blots, RT-PCR, please refer to Table S6.

### Parasite lines used and generated in this study

Please refer to Table S7 and www.pberghei.eu.

## Supporting Information

Protocol S1 Supplemental Methods
(0.06 MB DOC)

Figure S1 CAR-I/Trailer Hitch homolog CITH. ClustalW alignment of *Plasmodium berghei* CITH PB000768.03.0 (www.plasmodb.org) with homologs of *Drosophila melanogaster* (AAL39211.1; Trailer Hitch), human (Q9BX40  =  FAM61B) and *Caenorhabditis elegans* (NP_493254.1  =  CAR-1) recovered from BLASTP hits at www.ncbi.nlm.nih.gov. Identical and similar amino acids are indicated in black and grey shading, respectively.
(0.03 MB PDF)

Figure S2 Generation and characterisation of the mutant parasite (909cl1) expressing a *pb000768.03.0::gfp* fusion protein. (A) Schematic representation of the tagging plasmid for *pb000768.03.0*. Primers used for generating the targeting regions, and used in diagnostic PCRs are shown. Not drawn to scale. (B) Diagnostic PCRs showing correct 5′ and 3′ integration of the construct into the genomic locus; additional PCRs are shown for the tgdhfr/ts gene, the wild type gene and a control reaction. (C) FIGE analysis showing correct integration of the targeting plasmid into chromosome 13 of the parental parasite line 909. Hybridisation with a 3′UTR *P. berghei* dhfr/ts probe results in a signal in chromosome 7 of the endogenous dhfr/ts gene. (D) Northern blot of wild type and mutant gene fusion parasite clone showing normal wild type mRNA storage behaviour of translationally repressed mRNAs *p25* and *p28*. (E) RT-PCR analysis of transcripts from 909cl1 CITH::GFP (top panel) and wild type gametocytes (lower panel). Note the absence of wild type transcript in the mutant line and absence of the tagged transcript in the wild type parasite (lane 2775×2958 and 2775×1753). Positions of primers are shown (drawn to scale).
(0.11 MB PDF)

Figure S3 Correlation factors IP. Plots of (A) average ratio of peptide hits in 3 independent pull-down experiments and the molecular weight of the identified proteins. The Spearman correlation coefficient r = -0.076. (B) between average ratio of peptide hits in 3 independent pull-down experiments and number of peptide hits identified in the gametocyte-specific proteome (Khan *et al.* 2005). The Spearman correlation coefficient r = -0.188. No correlation was identified in either case.
(0.04 MB PDF)

Figure S4 Eukaryotic initiation factor 4E PB000857.02.0. ClustalW alignment of *Plasmodium berghei* eIF4E PB000857.02.0 (www.plasmodb.org) with homologs of *Drosophila melanogaster* (AAS93738.1), human (AAC39871.1  =  translation initiation factor 4E) and *Caenorhabditis elegans* (NP_499751.2  =  Initiation Factor 4E [eIF4E] family member) recovered from BLASTP hits at www.ncbi.nlm.nih.gov. Identical and similar amino acids are indicated in black and grey shading, respectively.
(0.02 MB PDF)

Figure S5 Poly(A) binding protein PB001286.00.0. ClustalW alignment of *Plasmodium berghei* PABP PB001286.00.0 (www.plasmodb.org) with homologs of *Drosophila melanogaster* (P21187  =  Polyadenylate-binding protein), human (CAI12300.1  =  poly(A) binding protein), *Caenorhabditis elegans* (NP_001021709.1  =  PolyA Binding protein family member [pab-1]) and *Saccharomyces cervisiae* (NP_011092.1  =  Pab1p) recovered from BLASTP hits at www.ncbi.nlm.nih.gov. Identical and similar amino acids are indicated in black and grey shading, respectively.
(0.03 MB PDF)

Figure S6 Homolog of Bruno (HoBo) PB001285.00.0. ClustalW alignment of *Plasmodium berghei* HoBo PB001285.00.0 (www.plasmodb.org) with homologs of *Drosophila melanogaster* (AAB58464.1  =  BRUNO), human (BAD93011.1  =  bruno-like 4 protein) and *Caenorhabditis elegans* (AAB37881.1  =  Elav-type RNA binding protein family protein 1) recovered from BLASTP hits at www.ncbi.nlm.nih.gov. Identical and similar amino acids are indicated in black and grey shading, respectively.
(0.03 MB PDF)

Figure S7 Homolog of Musashi (HoMu) PB000805.02.0. ClustalW alignment of *Plasmodium berghei* Musashi-like PB000805.02.0 (www.plasmodb.org) with homologs of *Drosophila melanogaster* (musashi; NP_733108.2), human (hnRNP A2/B1; EAW93834.1), *Caenorhabditis elegans* (musashi; NP_497799.1) and *Arabidopsis thaliana* (UBA2A; NP_567042.1) recovered from BLASTP hits at www.ncbi.nlm.nih.gov. Identical and similar amino acids are indicated in black and grey shading, respectively.
(0.03 MB PDF)

Figure S8 The ALBA domain proteins of *Plasmodium berghei* and selected Alveolata. (A) Partial sequence alignment (the highly divergent C-termini of the proteins shown have been omitted) of members belonging to the MDP2-like group. (B) Complete sequence alignment of members of the Rpp20-like group. Proteins were aligned using ClustalW at www.ch.embnet.org using default setting. The grey bar indicates the position of the Alba domain (PF01918) according to www.pfam.org. All protein identifiers (e.g. Q4Z4H5_PLABE) are from www.uniprot.org and indicate the following species: PLABE *Plasmodium berghei*, PLAF7 *Plasmodium falciparum*, TOXGO *Toxoplasma gondii*, 9ALVE *Perkinsus marinus*, BABBO *Babesia bovis*, THEPA *Theileria parvum*, 9CRYT *Cryptosporidium muris*.
(0.03 MB PDF)

Figure S9 Partial ClustalW alignment of *Plasmodium berghei* PGAM PB001107.03.0 (www.plasmodb.org) with orthologs of *P. chabaudi* (PC001355.02.), *P. falciparum* (PFC0430w), *P. knowlesi* (PKH_082990), *P. vivax* (PVX_119620) and *P. yoelii* (PY07389) recovered from BLASTP hits at www.plasmodb.org. Identical and similar amino acids are indicated in black and grey shading, respectively.
(0.05 MB PDF)

Figure S10 Enolase PB000456.03.0. ClustalW alignment of *Plasmodium berghei* Enolase PB000456.03.0 (www.plasmodb.org) with homologs of *Drosophila melanogaster* (P15007), human (NP_001966.1), *Caenorhabditis elegans* (NP_001022349.1) and yeast (P00924) recovered from BLASTP hits at www.ncbi.nlm.nih.gov. Identical and similar amino acids are indicated in black and grey shading, respectively.
(0.03 MB PDF)

Figure S11 Generation and characterisation of *pb000768.03.0 (cith)* gene null mutant parasite clone 856cl1. (A) Schematic organisation of the replacement construct for disruption of *pb000768.03.0*. The positions of primers used for generating the gene targeting regions for homologous recombination, and used in diagnostic PCRs are shown. Not drawn to scale. (B) Diagnostic PCRs showing correct integration of the plasmid into the genomic locus; additional PCRs include amplification of the tgdhfr/ts gene, the wild type gene and a control reaction. (C) FIGE analysis showing correct integration of the targeting plasmid into chromosome 13 of the parental population 856. Hybridisation with a 3′UTR *P. berghei* dhfr/ts probe results in a signal in chromosome 7 of the endogenous dhfr/ts gene, the signal in chromosome 2/3 is the *gfp* gene containing a dhfr/ts 3′UTR. (D) Northern analysis of wild type and null mutant parasite clone; no signal for *cith (pb000768.03.0)* is present in the mutant parasite clone 856cl1. Hybridisation to *rrna* and *p47* are used as loading control.
(0.04 MB PDF)

Figure S12 Generation and characterisation of *pb000768.03.0 (cith)* gene null mutant parasite clone 934cl1. (A) Schematic organisation of the KO targeting plasmid for *pb000768.03.0*. Primers used for generating the trageting regions, and used in diagnostic PCRs are shown. Not drawn to scale. (B) Diagnostic PCRs showing correct integration of the plasmid into the genomic locus; additional PCRs include amplification of the tgdhfr/ts gene, the wild type gene and a control reaction. (C) FIGE showing correct integration of the targeting plasmid into chromosome 13 of the parental population 934. Hybridisation with a 3′UTR *P. berghei* dhfr/ts probe results in a signal in chromosome 7 of the endogenous dhfr/ts gene, the signal in chromosome 2/3 is the *gfp* gene containing a dhfr/ts 3′UTR. (D) Northern blot of wild type and null mutant parasite clone. No signal for *pb000768.03.0* is present in the KO parasite clone. Input total RNA was controlled through hybridisation to *ribosomal RNA*. CITH KO parasites show destabilisation of otherwise abundant and repressed *p25* and *p28* mRNAs.
(0.04 MB PDF)

Figure S13 Generation and characterisation of *pb000603.01.0 (dozi)* gene null mutant parasite clone 927cl1. (A) Schematic organisation of the KO targeting plasmid for *pb000603.01.0*. Primers used for generating the trageting regions, and used in diagnostic PCRs are shown. Not drawn to scale. (B) Diagnostic PCRs showing 5′ and 3′ integration of the plasmid into the genomic locus; additional PCRs include amplification of the tgdhfr/ts gene, the wild type gene and a control reaction. (C) FIGE showing correct integration of the targeting plasmid into chromosome 12 of the parental population 927. Hybridisation with a 3′UTR *P. berghei* dhfr/ts probe results in a signal in chromosome 7 of the endogenous dhfr/ts gene, the signal in chromosome 2/3 is the *gfp* gene containing a dhfr/ts 3′UTR. (D) Northern blot of wild type and null mutant parasite clone. No signal for *pb000603.01.0* is present in the KO parasite clone. Input total RNA is controlled through hybridisation to *rrna*. DOZI KO parasites show destabilisation of otherwise abundant and repressed *p25* and *p28* mRNAs.
(0.04 MB PDF)

Figure S14 Generation and analysis of parasite reference line 820cl1m1cl1 that stably expresses GFP in male gametocytes and RFP in female gametcoytes. (A) Schematic representations of (1) the vector used to introduce the *gfp/rfp* male/female expression cassette into the *p230p* locus, (2) the *p230p* genomic locus, (3) the resulting integration in the genome of parasites after positive selection with pyrimethamine and (4) the genomic locus after negative selection with 5-fluorocytosine (5FC). Vector pL1186 is linearised at the *Ksp*I sites. Integration of pL1186 into the genome occurs by double cross-over homologous recombination resulting in a 1kb deletion of the non-essential *p230p* gene of parasites that are selected with pyrimethamine. After negative selection with 5FC, parasites are selected in which the positive/negative selectable marker cassette (hdfr-yfcu) has been excised from the integrated construct by a recombination event between the two *3′dhfr* sequences (blue chequered). Arrows indicate the position and size of expected restriction site fragments in Southern analysis (see B). (B) Genotype analysis of parasites after positive selection (line 820) and after negative selection from four mice (m1-m4). Southern analysis of separated chromosomes and restricted DNA shows the correct integration op pL1186 in the *p230p* locus on chromosome 3. Southern analysis *Pst*I/*Nco*I digested DNA of parasites after 5-FC treatment (m1-m4) show the presence of the GFP-positive DNA fragment with a reduced size (1.6 kb instead of 3.3 kb in line 820) after recombination has resulted in the excision of the selectable marker cassette. Parasites of 820cl1m1 were cloned by limiting dilution yielding line 820clm1cl1.
(0.05 MB PDF)

Figure S15 CITH and DOZI deletion mutant gametocytes suffer mRNA loss. (A) Dot plot of transcripts differentially regulated in DOZI and CITH KO mutants. (B) Gene ontology (GO) content according to *P. falciparum* orthologs of the commonly differentially expressed genes according to GO categories: biological process, cellular component and molecular function. Genes without GO assignment are shaded light blue. GO lists for *P. falciparum* are from AmiGO version August 2008.
(0.04 MB PDF)

Table S1 LC-MS/MS results of immunoprecipitation eluates using DOZI::GFP and CITH::GFP gametocyte lysates
(0.04 MB XLS)

Table S2 Microarray analysis of genes differentially regulated in the CITH-KO
(0.05 MB XLS)

Table S3 Microarray analysis of genes differentially regulated in DOZI-KO and CITH-KO
(0.04 MB XLS)

Table S4 Manually curated genes differentially regulated in CITH-KO and/or DOZI-KO gametocytes
(0.03 MB XLS)

Table S5 The *Plasmodium falciparum* and *P. berghei* complement of proteins involved in RNA degradation
(0.04 MB XLS)

Table S6 Oligonucleotide primer list
(0.02 MB XLS)

Table S7 *Plasmodium berghei* transgenic parasite lines
(0.02 MB XLS)
