# Supplementary material for: Universal Features of Post-Transcriptional Gene Regulation Are Critical for Plasmodium Zygote Development
Source: PLoS Pathog. 2010 Feb 12;6(2):e1000767. doi: 10.1371/journal.ppat.1000767 (PMC2820534; doi:10.1371/journal.ppat.1000767)
Supplement: Protocol S1 — Supplemental Methods (0.06 MB DOC) [file ppat.1000767.s001.doc]

**Protocol S1**

**Mass-spectrometric analysis of immunoprecipitation eluates.** Immunoprecipitation eluates were processed following (Lasonde*r et* al. 2002) and peptide mass spectrometric experiments performed using a nano-HPLC Agilent 1100 system connected to a 7-Tesla linear quadrupole ion trap-Ion Cyclotron Resonance Fourier transform (LTQ-FT) mass spectrometer (Thermo Fisher). Peptides were separated on 15 cm 100 µm ID PicoTip (New Objective) columns packed with 3µm Reprosil C18 beads (Dr. Maisch GmbH) using a 45 min gradient from 10% buffer B to 35% buffer B (80% acetonitrile in 0.5% acetic acid). Peptides eluting from the column tip were electrosprayed directly into the mass spectrometer with a spray voltage of 2.1 kV. Peptide selection and fragmentation was set by the Xcalibur 1.4 data acquisition software (Thermo Fisher). The mass spectrometer was operated in the data-dependent mode to sequence the four most intense ions per duty cycle. Briefly, full-scan MS spectra of intact peptides (m/z 350–1500) with an automated gain control accumulation target value of 1E6 ions were acquired in the Fourier transform ion cyclotron resonance (FT ICR) cell with a resolution of 50,000. The 4 most abundant ions were sequentially isolated and fragmented in the linear ion trap by applying collisionally induced dissociation using an accumulation target value of 20,000 (capillary temperature, 150°C; normalized collision energy, 27%). A dynamic exclusion of ions previously sequenced within 180s was applied. All unassigned charge states were excluded from sequencing. A minimum of 500 counts was required for MS2 selection. RAW spectrum files were converted into a Mascot generic peaklist using DTA Supercharger (http://msquant.sourceforge.net). Proteins were identified by searching peak lists containing fragmentation spectra with Mascot version 2.1 (Matrix Science) against an in-house *P. berghei* PlasmoDB version5.0 supplemented with the protein sequences of mouse International Protein Index (IPI) database version 3.16. Mascot search parameters for protein identification specified an initial mass tolerance of 30 ppm for the parental peptide and 0.8 Da for fragmentation spectra and a trypsin enzyme specificity allowing up to 3 miscleaved sites. Carbamidomethylation of cysteines was specified as a fixed modification, oxidation of methionines, and deamidation of glutamine or asparagine were set as variable modifications.

Internal mass calibration of measured ions was performed simultaneously with parsing Mascot search result html files using MSQuant open-source software ([www.msquant.sourceforge.net](http://www.msquant.sourceforge.net/)) into text files. A final absolute mass tolerance for the parental peptide was determined at 10 ppm. For assembling final peptide and protein lists, general filtering criteria were applied which were developed by in-house reverse database searches for large scale *P. falciparum* and human datasets. Multiple charged peptides with precursor masses larger than 350 and a minimal Mascot peptide score of 23 were required for proteins identified by 3 or more non-redundant peptides. More stringent criteria were required to identify proteins sequenced by two and one unique peptide per protein with high confidence. A false-positive rate of 0.13 % was determined for proteins identified by 2 unique unmodified peptides per LC MS/MS analysis. A false-positive rate of 1.7 % was obtained for proteins identified by 1 unmodified peptide with a peptide cut off score of 40, and a Mascot peptide delta score of 10. Pull-down specificity was determined as the ratio of unique number of peptide hits in the anti-GFP pull down and unique number of peptide hits in the anti-cmyc IP control as follows: specificity = (nGFP+1)/(ncmyc+1) – 1 and detected in DOZI and CITH IPs.

**Generation of a reporter *P. berghei* line (820cl1m1cl1) that expresses RFP in female gametocytes, gametes and zygotes.** To analyse fertilisation and meiosis by FACS we generated a reporter line that expresses RFP in females and GFP in males. This line was constructed with a plasmid construct that contains a) a male GFP expression cassette, b) a female RFP expression cassette and c) a drug selection cassette containing a fusion of the positive selectable marker *hdhfr* and the negative selectable marker *yfcu* (Brak*s et* al. 2006).

a) *Male GFP expression cassette*: The *green fluorescent protein mutant 3* (*gfpm3*) gene (*Bam*HI/*Xba*I) of pL0017 ([www.mr4.org](http://www.mr4.org/)) was exchanged for the *e-gfp* gene of plasmid pEGFP-NI (Clontech, subcloned *Sac*II/*Not*I in pBluescript-SK). Then the *3’utr* *pbdhfr/ts* (*3’dhfr*) of pL0017eGFP was replaced by the *3’utr* of the *P. berghei calmodulin* gene (3’*cam*, 2566-*Asp*718I and 2567-*Xba*I). Next, the *eef1a* promoter was replaced by the male specific promoter of *pb000791.03.0* from plasmid pL0012 (1531 bp *Eco*RV/*Bam*HI fragment (Kha*n et* al. 2005).

b) *Female RFP expression cassette*: The *3’ utr* of *pbdhfr* in pL1102 was replaced with the *3’ utr* of *p48/45* (*3’ 48/45*; *Asp*718/*Xba*I; 1882-*Asp*718I and 1881-*Xba*I). The *eef1a* promoter was replaced with the 1151 bp female specific promoter of *ccp2* (*pb000504.02.0*) of plasmid pL1118 (*Eco*RV/*Bam*HI fragment (Kha*n et* al. 2005)). Finally the RFP expression cassette (*5’ccp2-rfp-3’48/45*) was cloned into pBluescript-SK (EcoRV/Asp718) to create pBSFeRFP. In this plasmid the Asp718I fragment of the male GFP expression cassette (*5’ pb000791.03.0-egfp-3’cam*) was cloned resulting in plasmid pBSFeRFP-MaGFP.

c) *Drug-selection cassette:* The *eef1aa-gfp-3’dhfr* cassette of pL0023 ([www.mr4.org](http://www.mr4.org/)) was replaced by the *amaI-gfp-3’dhfr* cassette of pBSSKamaI-gfp-3’UTR (Franke-Fayar*d et* al. 2008) to obtain pL1141. Subsequently, the 230p integration cassette of pL1141 (*Asp*178I/*Not*I blunt) was introduced in pL0035 that contains the fusion gene of *hdfr* and *yfcu* (*Asp*718I/*Eco*RV) (Brak*s et* al. 2006) to create plasmid FCU230p. The *Asp*718I fragment of the male/female expression cassette of pBSFeRFP-MaGFP was cloned into plasmid FCU230p to make pL1186 (see Figure S14 for a schematic representation of pL1186). We used the *hdfr-yfcu* selection cassette from pL0035 to be able to remove the complete drug selectable marker by negative selection as described (Brak*s et* al. 2006). Plasmids and sequences of pL0017, pL0023, pL0035 and pL1102 are available from MR4 ([www.mr4.org](http://www.mr4.org/) ).

Parasites of the ANKA strain cl15cy1were transfected with pL1186 and mutant parasites selected with pyrimethamine (Jans*e et* al. 2006) resulting in line 820. Before applying negative selection, parasites were cloned by limiting dilution and we selected 820cl1 for further analysis. Correct integration of construct pL1186 in 820cl1 was shown by diagnostic PCRs and Southern analysis of digested genomic DNA and separated chromosomes (Figure S14). Negative selection with 5-fluorocytosine (5-FC) was as described (Brak*s et* al. 2006); 4 mice infected with 820cl1 were treated with 5-FC starting at a parasitemia of 0.1-0.5% with a daily single dose of 0.5 ml of a solution of 20 mg/ml a day for a period of 4 days. Resistant parasites were collected between days 5-7 after start of the 5-FC treatment and the genotype analyzed by diagnostic Southern analysis to confirm removal of the drug-selectable marker *hdfr-yfcu* by a recombination event between the two *3’ utr dhfr* sequences (see Figure S14). Parasites from one of the four mice (mouse 1) that had been treated with 5-FC were cloned by limiting dilution, resulting in line 820cl1m1cl1. Exclusive expression of the reporter proteins GFP and RFP in male and females gametocytes respectively and not in asexual stages was confirmed by fluorescence microscopy of live blood stage parasites obtained from the tail blood of infected mice and of purified gametocytes (Figure 4).

**Reference List**

Braks, J.A., Franke-Fayard, B., Kroeze, H., Janse, C.J., and Waters, A.P. 2006. Development and application of a positive-negative selectable marker system for use in reverse genetics in *Plasmodium*. *Nucleic acids research* **34**(5): e39.

Franke-Fayard, B., Djokovic, D., Dooren, M.W., Ramesar, J., Waters, A.P., Falade, M.O., Kranendonk, M., Martinelli, A., Cravo, P., and Janse, C.J. 2008. Simple and sensitive antimalarial drug screening in vitro and *in vivo* using transgenic luciferase expressing *Plasmodium* *berghei* parasites. *International journal for parasitology* **38**(14): 1651-1662.

Janse, C.J., Franke-Fayard, B., Mair, G.R., Ramesar, J., Thiel, C., Engelmann, S., Matuschewski, K., van Gemert, G.J., Sauerwein, R.W., and Waters, A.P. 2006. High efficiency transfection of *Plasmodium* *berghei* facilitates novel selection procedures. *Molecular and biochemical parasitology* **145**(1): 60-70.

Khan, S.M., Franke-Fayard, B., Mair, G.R., Lasonder, E., Janse, C.J., Mann, M., and Waters, A.P. 2005. Proteome analysis of separated male and female gametocytes reveals novel sex-specific *Plasmodium* biology. *Cell* **121**(5): 675-687.

Lasonder, E., Ishihama, Y., Andersen, J.S., Vermunt, A.M., Pain, A., Sauerwein, R.W., Eling, W.M., Hall, N., Waters, A.P., Stunnenberg, H.G., and Mann, M. 2002. Analysis of the *Plasmodium* *falciparum* proteome by high-accuracy mass spectrometry. *Nature* **419**(6906): 537-542.
