# Supplementary material for: Universal Features of Post-Transcriptional Gene Regulation Are Critical for Plasmodium Zygote Development
Source: PLoS Pathog. 2010 Feb 12;6(2):e1000767. doi: 10.1371/journal.ppat.1000767 (PMC2820534; doi:10.1371/journal.ppat.1000767)
Supplement: Figure S1 — CAR-I/Trailer Hitch homolog CITH. ClustalW alignment of Plasmodium berghei CITH PB000768.03.0 (www.plasmodb.org) with homologs of Drosophila melanogaster (AAL39211.1; Trailer Hitch), human (Q9BX40 = FAM61B) and Caenorhabditis elegans (NP_493254.1 = CAR-1) recovered from BLASTP hits at www.ncbi.nlm.nih.gov. Identical and similar amino acids are indicated in black and grey shading, respectively. (0.03 MB PDF) [file ppat.1000767.s002.pdf]

```
C.elegans     1 --MSNQTPYIGSKISLISKLDIRYEGILYTVDTNDSTIALAKVRSFGTEK
Drosophila    1 --MSGGLPELGSKISLISKADIRYEGRLYTVDPQECTIALSSVRSFGTED
Homo          1 MSGSSGTPYLGSKISLISKAQIRYEGILYTIDTDNSTVALAKVRSFGTED
P.berghei     1 MSSVSTLPYIGSKISLISNSEIRYEGILYTINTHESTVALQNVRSYGTEG

C.elegans    49 RPTANPVAARDDVYEYIIFKASDIKDLIVCD-------------------
Drosophila   49 RDTQFQIAPQSQIYDYILFRGSDIKDIRVVNNHTLPHHNDPAIMQAQLQN
Homo         51 RPTDRPAPPREEIYEYIIFRGSDIKDITVCE---------PPKAQHTLPQ
P.berghei    51 R-RQPDIPASNEVYDFIIFRGKDIKDVTVSEAP------------KTIPD

C.elegans    80 ----TPKMANIGGGLPYDPAIISVSSRSAP------ASDGAPAASAGSSR
Drosophila   99 GPPQMPQHFPMPSGMSGPPQQQQVPSQQPPQMPSGGGSGGAGGAGAPGGG
Homo         92 DPAIVQSSLGSASASPFQPHVPYSPFRGMAPYGPLAASSLLSQQYAASLG
P.berghei    88 DPAIVSMNIAPSTKNNIGDNLNYNNNVNNINKPLNVQNNMIPQNERNVNL

C.elegans   120 AGTPSRNSPLG---------------------------------------
Drosophila  149 GPGYGNGNPFGNLGGPNLANMVGNAGGSLAPGSGAPGSGPFMHIGGNQQQ
Homo        142 LGAGFPSIPVGKSPMVEQAVQTGSADN-----------------------
P.berghei   138 NNRRYYNRHN----------------------------------------

C.elegans   131 ------------------------------------QIIQNQRPGRGG--
Drosophila  199 PKPQQQKQPNMLAGASRSTTPISLIVSPTAELTQQQQLHQQQNAGQGGNG
Homo        169 ------------------------------------LNAKKLLPGKGTTG
P.berghei   148 -----------------------------------YNYYYNGNIGNGNMN

C.elegans   143 --------------------------------------------------
Drosophila  249 RDAGHKRQNHQQQQNQQHQQRGGPSHNNMQQQQQRGGGSGTDFYNQQRDR
Homo        183 TQLNGRQAQPSSKTASDVVQP-----------------------------
P.berghei   163 SNAN----------------------------------------------

C.elegans   143 ----------YQQNFQANRGYNNYRGGFAGGYNNQRGHNN----------
Drosophila  299 RDSGRQMDNNYSNNYNNNNNNQRNRGGGNGMQQQQRGGNGGSGGGGGNGG
Homo        204 -----AAVQAQGQVNDENRRPQRRRSGNRRTRNRSRGQN-----------
P.berghei   167 ----------HNNNHNNNRHYNNFKYKNYRNYDRQSYVIG----------

C.elegans   173 -----------------------------------------YGVPRVNHR
Drosophila  349 GNNPAWNMHRGNQNSNNMMNMRNRGMGSRGPMRPNQGYRPQSANNQNKPR
Homo        238 -------------------------------------------RPTNVKE
P.berghei   197 -----------------------------------------ELESQPNPV

C.elegans   182 EKLKFESDFDFEKANEKF--QEVLVDNLEKLNIEDKAEP-----------
Drosophila  399 NKIKFEGDFDFEQANNKF--EELRSQLAKLKVAEDGAPKPATNATAATAT
Homo        245 NTIKFEGDFDFESANAQFNREELDKEFKKKLNFKDDKAEKGEEKDLAVVT
P.berghei   206 LKSKFSPDFDFSSNNLKFDKTNIIDEKSKDVLSAN---------------

C.elegans   219 -----------------------------------EVEEKKDAAFYDKKT
Drosophila  447 ATNEQVGEKVEGVHTLNGETDKKDDSGNETGAGEHEPEEDDVAVCYDKTK
Homo        295 QSAEAP----------------------------AEEDLLGPNCYYDKSK
P.berghei   241 --------------------------------------SNIQVGGYDKKS

C.elegans   234 SFFDNISCESLEKAEGKTGRPDWKKERETNQETFGHNAVRSLNYRRG---
Drosophila  497 SFFDNISCEAAQDRS-KNKKNDWRQERKLNTETFGVSSTRRGSYRGRNQY
Homo        317 SFFDNISSELKTS----SRRTTWAEERKLNTETFGVSGRFLR--------
P.berghei   253 SFFDNISCETLDKQQGKDEKVDREKLRMLDVDTFGIAAAHYRTNMHNRNN

C.elegans   281 FGGRGRGGNRGYGGYNNGYQHQHQHRGGYNGGYR-----QNNGGYRRGGY
Drosophila  546 YNNNGNGGINSGYGGAPGYNRNNYRMGGGGGNFRNRSNNRNNGGGRGGNG
Homo        355 -GRSSRGGFRGGRGNGTTRRNPTSHRAGTGRV------------------
P.berghei   303 NRNKGRNSKNNKMMGNFNYNYYNRNQNPFNRYPAY---------------

C.elegans   326 APRDNQGNTAAAAEQ-----------------------------------
Drosophila  596 MPNITNGNTAAALKAANNAAGHGSNATDSSAPNATTATTKSTSLLPEQTQ
Homo            --------------------------------------------------
P.berghei       --------------------------------------------------
```
