# Supplementary figures and images for: Universal Features of Post-Transcriptional Gene Regulation Are Critical for Plasmodium Zygote Development
Source: PLoS Pathog. 2010 Feb 12;6(2):e1000767. doi: 10.1371/journal.ppat.1000767 (PMC2820534; doi:10.1371/journal.ppat.1000767)

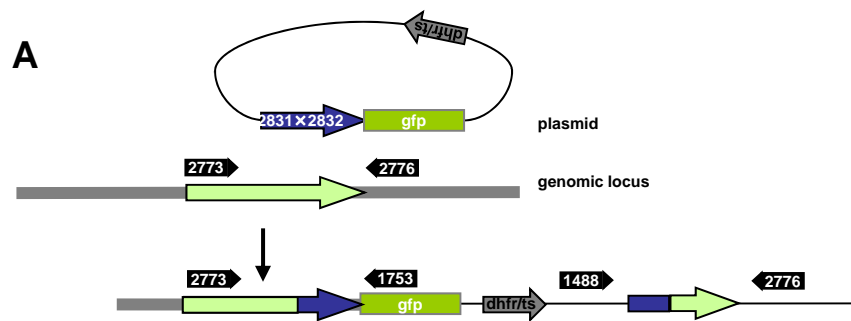

5' targeting region  
3' targeting region  
gene targeted for KO  
primers

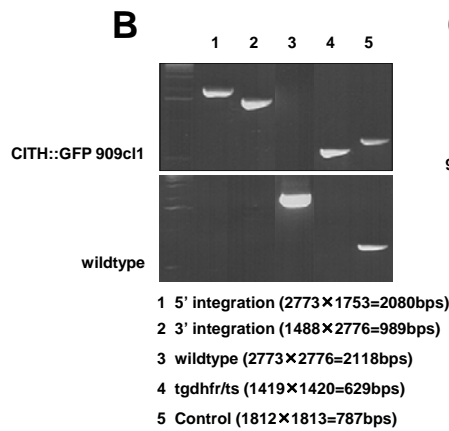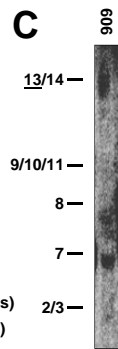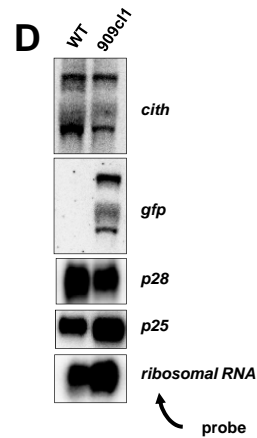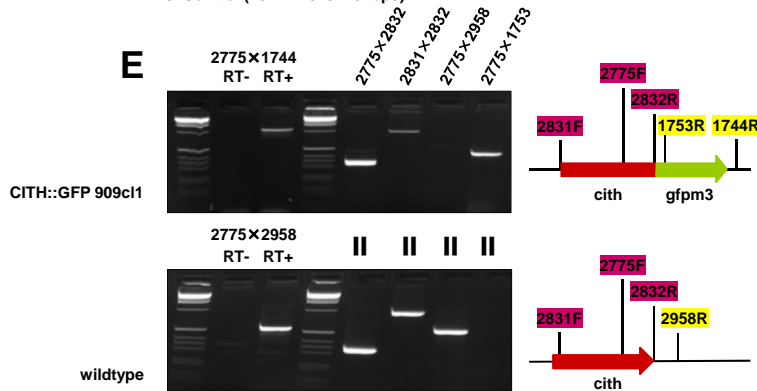

Supplement: Figure S2 — Generation and characterisation of the mutant parasite (909cl1) expressing a pb000768.03.0::gfp fusion protein. (A) Schematic representation of the tagging plasmid for pb000768.03.0. Primers used for generating the targeting regions, and used in diagnostic PCRs are shown. Not drawn to scale. (B) Diagnostic PCRs showing correct 5′ and 3′ integration of the construct into the genomic locus; additional PCRs are shown for the tgdhfr/ts gene, the wild type gene and a control reaction. (C) FIGE analysis showing correct integration of the targeting plasmid into chromosome 13 of the parental parasite line 909. Hybridisation with a 3′UTR P. berghei dhfr/ts probe results in a signal in chromosome 7 of the endogenous dhfr/ts gene. (D) Northern blot of wild type and mutant gene fusion parasite clone showing normal wild type mRNA storage behaviour of translationally repressed mRNAs p25 and p28. (E) RT-PCR analysis of transcripts from 909cl1 CITH::GFP (top panel) and wild type gametocytes (lower panel). Note the absence of wild type transcript in the mutant line and absence of the tagged transcript in the wild type parasite (lane 2775×2958 and 2775×1753). Positions of primers are shown (drawn to scale). (0.11 MB PDF) [file ppat.1000767.s003.pdf]

## A molecular weight vs IP MS

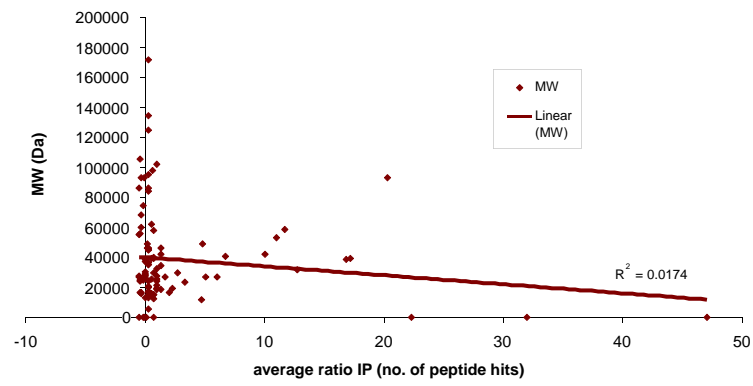

## B gametocyte MS vs IP MS

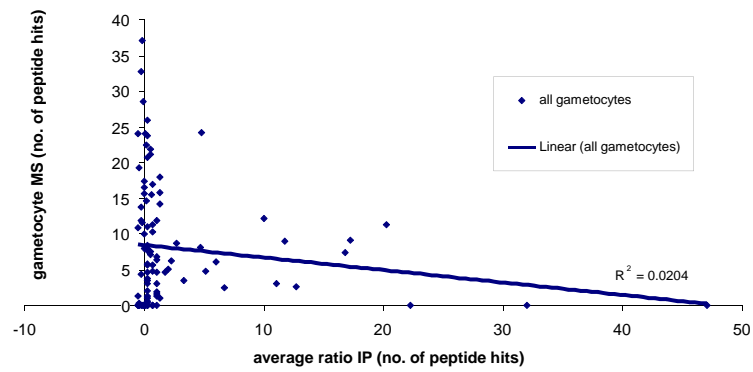

Supplement: Figure S3 — Correlation factors IP. Plots of (A) average ratio of peptide hits in 3 independent pull-down experiments and the molecular weight of the identified proteins. The Spearman correlation coefficient r = -0.076. (B) between average ratio of peptide hits in 3 independent pull-down experiments and number of peptide hits identified in the gametocyte-specific proteome (Khan et al. 2005). The Spearman correlation coefficient r = -0.188. No correlation was identified in either case. (0.04 MB PDF) [file ppat.1000767.s004.pdf]

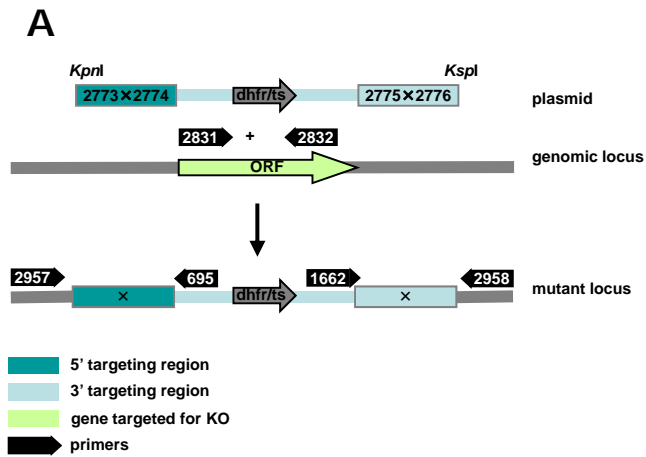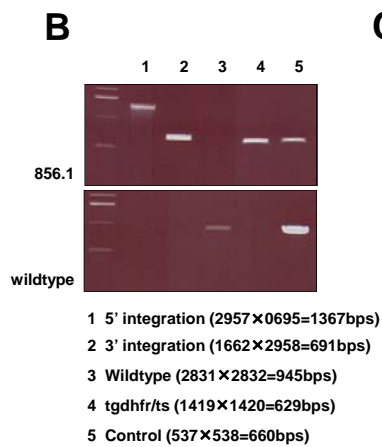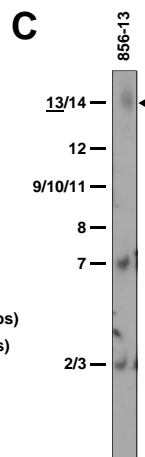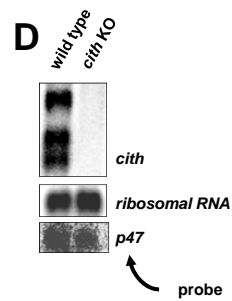

Supplement: Figure S11 — Generation and characterisation of pb000768.03.0 (cith) gene null mutant parasite clone 856cl1. (A) Schematic organisation of the replacement construct for disruption of pb000768.03.0. The positions of primers used for generating the gene targeting regions for homologous recombination, and used in diagnostic PCRs are shown. Not drawn to scale. (B) Diagnostic PCRs showing correct integration of the plasmid into the genomic locus; additional PCRs include amplification of the tgdhfr/ts gene, the wild type gene and a control reaction. (C) FIGE analysis showing correct integration of the targeting plasmid into chromosome 13 of the parental population 856. Hybridisation with a 3′UTR P. berghei dhfr/ts probe results in a signal in chromosome 7 of the endogenous dhfr/ts gene, the signal in chromosome 2/3 is the gfp gene containing a dhfr/ts 3′UTR. (D) Northern analysis of wild type and null mutant parasite clone; no signal for cith (pb000768.03.0) is present in the mutant parasite clone 856cl1. Hybridisation to rrna and p47 are used as loading control. (0.04 MB PDF) [file ppat.1000767.s012.pdf]

**A**

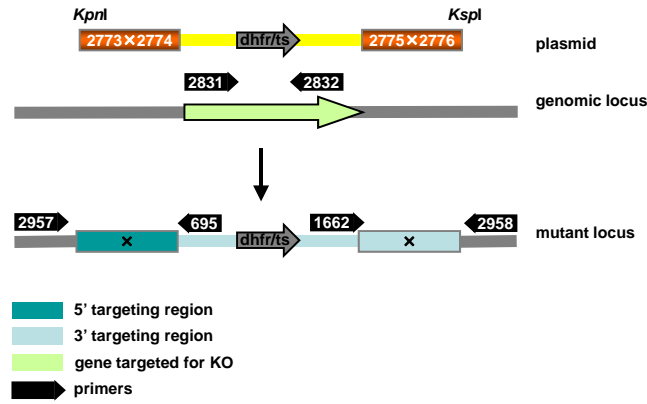

**B**

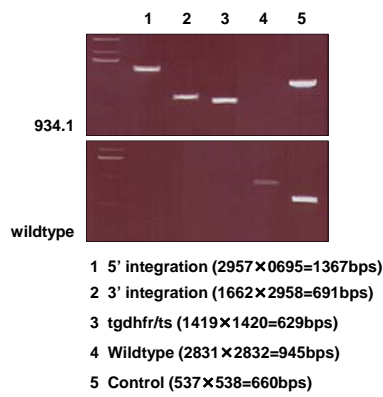

**C**

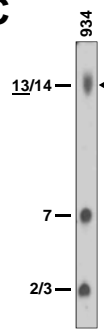

**D**

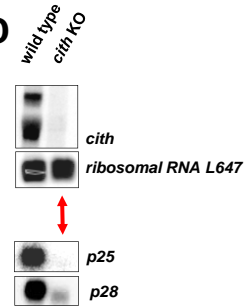

Supplement: Figure S12 — Generation and characterisation of pb000768.03.0 (cith) gene null mutant parasite clone 934cl1. (A) Schematic organisation of the KO targeting plasmid for pb000768.03.0. Primers used for generating the trageting regions, and used in diagnostic PCRs are shown. Not drawn to scale. (B) Diagnostic PCRs showing correct integration of the plasmid into the genomic locus; additional PCRs include amplification of the tgdhfr/ts gene, the wild type gene and a control reaction. (C) FIGE showing correct integration of the targeting plasmid into chromosome 13 of the parental population 934. Hybridisation with a 3′UTR P. berghei dhfr/ts probe results in a signal in chromosome 7 of the endogenous dhfr/ts gene, the signal in chromosome 2/3 is the gfp gene containing a dhfr/ts 3′UTR. (D) Northern blot of wild type and null mutant parasite clone. No signal for pb000768.03.0 is present in the KO parasite clone. Input total RNA was controlled through hybridisation to ribosomal RNA. CITH KO parasites show destabilisation of otherwise abundant and repressed p25 and p28 mRNAs. (0.04 MB PDF) [file ppat.1000767.s013.pdf]

**A**

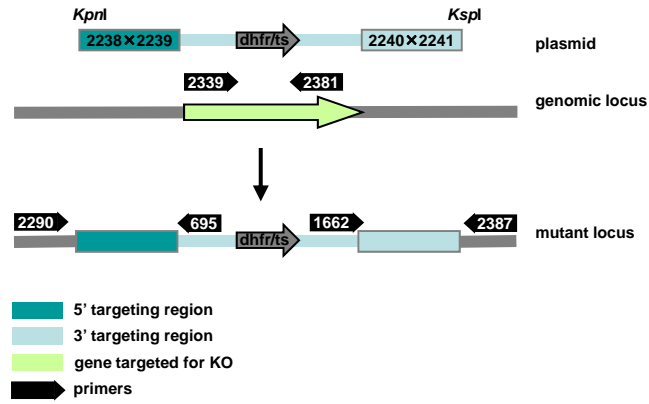

**B**

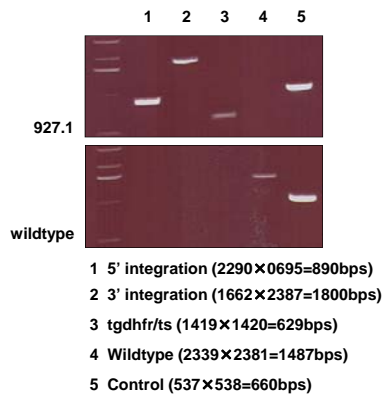

**C**

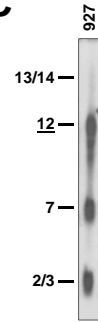

**D**

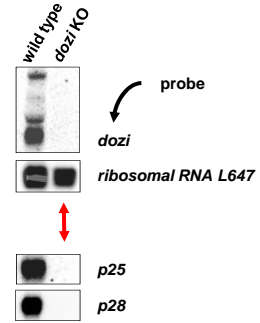

Supplement: Figure S13 — Generation and characterisation of pb000603.01.0 (dozi) gene null mutant parasite clone 927cl1. (A) Schematic organisation of the KO targeting plasmid for pb000603.01.0. Primers used for generating the trageting regions, and used in diagnostic PCRs are shown. Not drawn to scale. (B) Diagnostic PCRs showing 5′ and 3′ integration of the plasmid into the genomic locus; additional PCRs include amplification of the tgdhfr/ts gene, the wild type gene and a control reaction. (C) FIGE showing correct integration of the targeting plasmid into chromosome 12 of the parental population 927. Hybridisation with a 3′UTR P. berghei dhfr/ts probe results in a signal in chromosome 7 of the endogenous dhfr/ts gene, the signal in chromosome 2/3 is the gfp gene containing a dhfr/ts 3′UTR. (D) Northern blot of wild type and null mutant parasite clone. No signal for pb000603.01.0 is present in the KO parasite clone. Input total RNA is controlled through hybridisation to rrna. DOZI KO parasites show destabilisation of otherwise abundant and repressed p25 and p28 mRNAs. (0.04 MB PDF) [file ppat.1000767.s014.pdf]

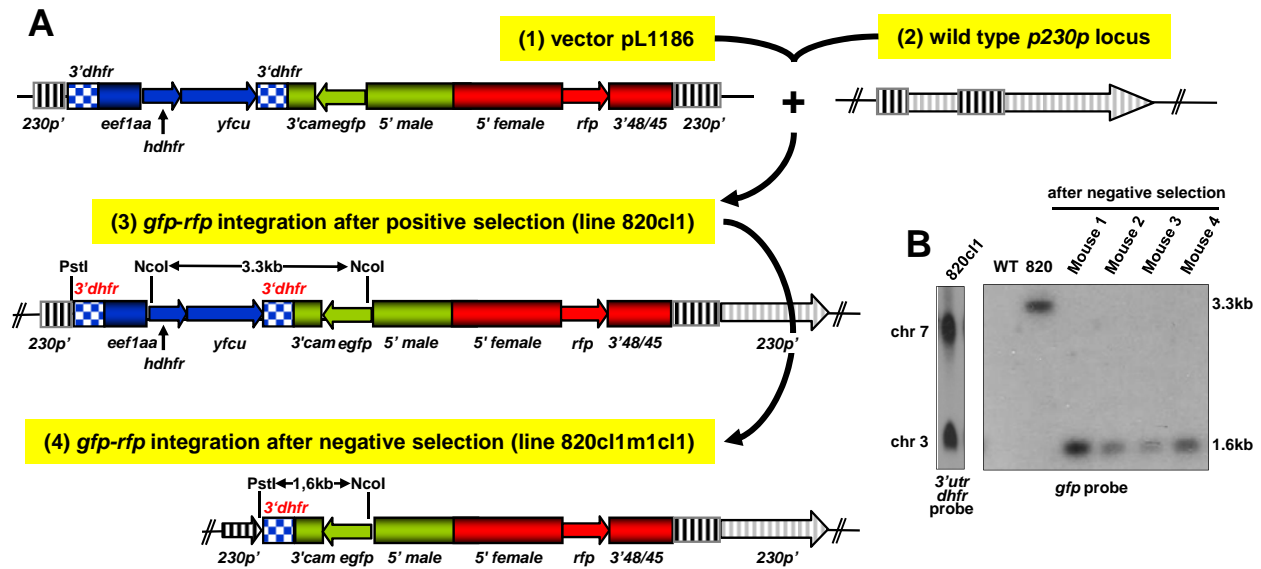

Supplement: Figure S14 — Generation and analysis of parasite reference line 820cl1m1cl1 that stably expresses GFP in male gametocytes and RFP in female gametcoytes. (A) Schematic representations of (1) the vector used to introduce the gfp/rfp male/female expression cassette into the p230p locus, (2) the p230p genomic locus, (3) the resulting integration in the genome of parasites after positive selection with pyrimethamine and (4) the genomic locus after negative selection with 5-fluorocytosine (5FC). Vector pL1186 is linearised at the KspI sites. Integration of pL1186 into the genome occurs by double cross-over homologous recombination resulting in a 1kb deletion of the non-essential p230p gene of parasites that are selected with pyrimethamine. After negative selection with 5FC, parasites are selected in which the positive/negative selectable marker cassette (hdfr-yfcu) has been excised from the integrated construct by a recombination event between the two 3′dhfr sequences (blue chequered). Arrows indicate the position and size of expected restriction site fragments in Southern analysis (see B). (B) Genotype analysis of parasites after positive selection (line 820) and after negative selection from four mice (m1-m4). Southern analysis of separated chromosomes and restricted DNA shows the correct integration op pL1186 in the p230p locus on chromosome 3. Southern analysis PstI/NcoI digested DNA of parasites after 5-FC treatment (m1-m4) show the presence of the GFP-positive DNA fragment with a reduced size (1.6 kb instead of 3.3 kb in line 820) after recombination has resulted in the excision of the selectable marker cassette. Parasites of 820cl1m1 were cloned by limiting dilution yielding line 820clm1cl1. (0.05 MB PDF) [file ppat.1000767.s015.pdf]

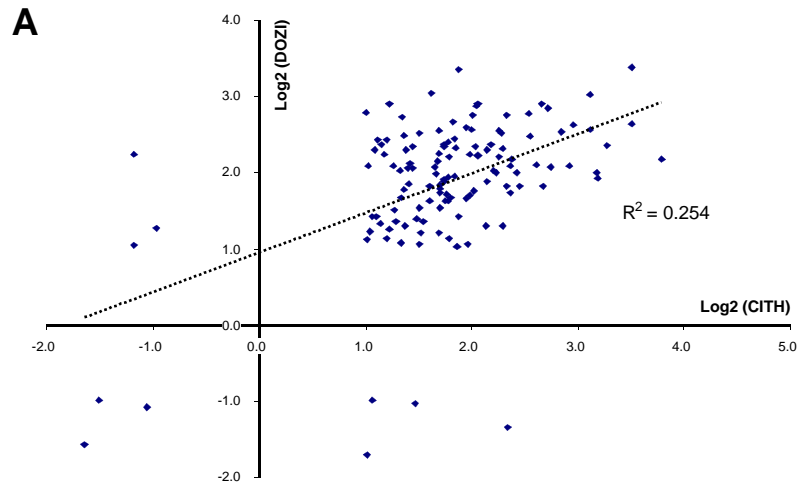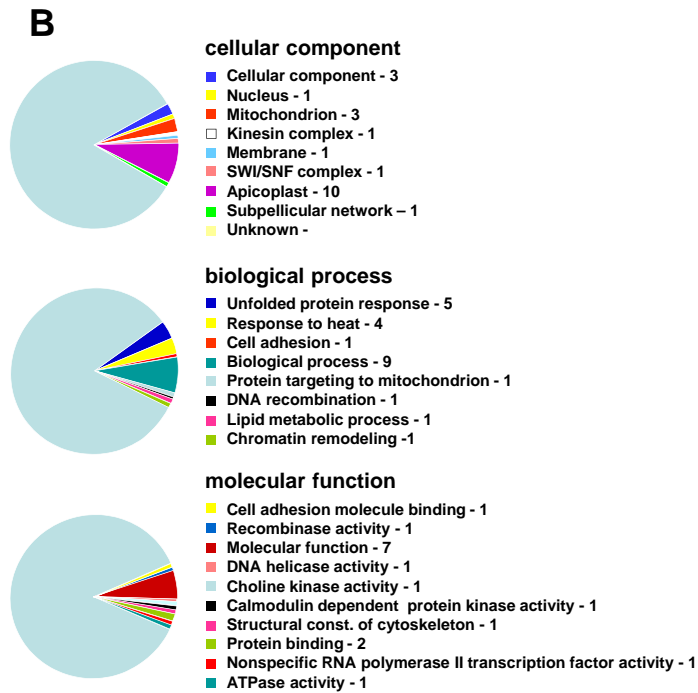

Supplement: Figure S15 — CITH and DOZI deletion mutant gametocytes suffer mRNA loss. (A) Dot plot of transcripts differentially regulated in DOZI and CITH KO mutants. (B) Gene ontology (GO) content according to P. falciparum orthologs of the commonly differentially expressed genes according to GO categories: biological process, cellular component and molecular function. Genes without GO assignment are shaded light blue. GO lists for P. falciparum are from AmiGO version August 2008. (0.04 MB PDF) [file ppat.1000767.s016.pdf]
