# Supplementary material for: Universal Features of Post-Transcriptional Gene Regulation Are Critical for Plasmodium Zygote Development
Source: PLoS Pathog. 2010 Feb 12;6(2):e1000767. doi: 10.1371/journal.ppat.1000767 (PMC2820534; doi:10.1371/journal.ppat.1000767)
Supplement: Figure S4 — Eukaryotic initiation factor 4E PB000857.02.0. ClustalW alignment of Plasmodium berghei eIF4E PB000857.02.0 (www.plasmodb.org) with homologs of Drosophila melanogaster (AAS93738.1), human (AAC39871.1 = translation initiation factor 4E) and Caenorhabditis elegans (NP_499751.2 = Initiation Factor 4E [eIF4E] family member) recovered from BLASTP hits at www.ncbi.nlm.nih.gov. Identical and similar amino acids are indicated in black and grey shading, respectively. (0.02 MB PDF) [file ppat.1000767.s005.pdf]

```
S.cerevisiae    1 ---------------------------------MSVEEVSKKFEENVSVD
Drosophila      1 MVVLETEKTSAPSTEQGRPEPPTSAAAPAEAKDVKPKEDPQETGEPAGNT
C.elegans       1 ----------------------------------------------MTET
Homo            1 -------------------------MMTVGTMIRMKKTAHRKIGEKEKTE
P.berghei       1 ---------------------------------------MKYLTFNKSSK

S.cerevisiae   18 DTTATPKTVLSDSAHFDVKHPLNTKWTLWYTKPAVDK---SESWSDLLRP
Drosophila     51 ATTTAPAGDDAVRTEHLYKHPLMNVWTLWYLE--NDR---SKSWEDMQNE
C.elegans       5 EQTTAP------------IYPLKRNWTWWYLN--DER---NKSWEDRLKK
Homo           26 RDKNQSSSKRKAVVPGPAEHPLQYNYTFWYSRRTPGRPTSSQSYEQNIKQ
P.berghei      12 DAFDLNEKIEATKIDLSNPLLLQYNWVIWEQVSDNKIKQ-SNNYKDYTRP

S.cerevisiae   65 VTSFQTVEEFWAIIQNIPEPHELPLKS---------------DYHVFRND
Drosophila     96 ITSFDTVEDFWSLYNHIKPPSEIKLGS---------------DYSLFKKN
C.elegans      38 VYTFNTVSEFWALYDAIRPPSGLNALC---------------DYNVFRDD
Homo           76 IGTFASVEQFWRFYSHMVRPGDLTGHS---------------DFHLFKEG
P.berghei      61 LAKFNSVQKFWQLWNRLPQPSDLLAQRSMTRFSDDGIFRIVDALMIFRDN

S.cerevisiae  100 VRPEWEDEANAKGGKWSFQLR--GKG-ADIDELWLRTLLAVIGETIDEDD
Drosophila    131 IRPMWEDAANKQGGRWVITLN--KSSKTDLDNLWLDVLLCLIGEAFDHSD
C.elegans      73 IQPMWEVPENSNGGRWLIVID-KGKTPEMVDAIWLEILMALVGEQFGKDM
Homo          111 IKPMWEDDANKNGGKWIIRLR-KG----LASRCWENLILAMLGEQFMVG-
P.berghei     111 IQPMWEDPANAGGGHFEYKILPKDFPYSQIDEFWNNLVLAIIGCSLKHYD

S.cerevisiae  147 SQING----VVLSIRKGGNKFALWTKS-EDKEPLLRIGGKFKQVLKLTDD
Drosophila    179 -QICG----AVINIRGKSNKISIWTADGNNEEAALEIGHKLRDALRLGRN
C.elegans     122 ESICG----LVCNVRGKGSKISVWTKDCNDDETNMRIGVVLKEKLMAASK
Homo          155 EEICG----AVVSVRFQEDIISIWNKTASDQATTARIRDTLRRVLNLPPN
P.berghei     161 LITGIRLVDKLSTTRYGYIRIEIWYTTITDENVRNHLRKDLEEHMCNRID

S.cerevisiae  192 GH-------LEFFPHSSANGRHPQPSITL----------------
Drosophila    224 NS-------LQYQLHKDTMVKQGSNVKSIYTL-------------
C.elegans     168 DHSKPLFDVIRYEDHESCQKKTSSVVKAKLSLHSSDAPVAEKSAV
Homo          201 -------TIMEYKTHTDSIKMPGRLGPQRLLFQNLWKPRLNVP--
P.berghei     211 GS-------HVYPPRVKSLSHVHK---------------------
```
