# Supplementary material for: Universal Features of Post-Transcriptional Gene Regulation Are Critical for Plasmodium Zygote Development
Source: PLoS Pathog. 2010 Feb 12;6(2):e1000767. doi: 10.1371/journal.ppat.1000767 (PMC2820534; doi:10.1371/journal.ppat.1000767)
Supplement: Figure S5 — Poly(A) binding protein PB001286.00.0. ClustalW alignment of Plasmodium berghei PABP PB001286.00.0 (www.plasmodb.org) with homologs of Drosophila melanogaster (P21187 = Polyadenylate-binding protein), human (CAI12300.1 = poly(A) binding protein), Caenorhabditis elegans (NP_001021709.1 = PolyA Binding protein family member [pab-1]) and Saccharomyces cervisiae (NP_011092.1 = Pab1p) recovered from BLASTP hits at www.ncbi.nlm.nih.gov. Identical and similar amino acids are indicated in black and grey shading, respectively. (0.03 MB PDF) [file ppat.1000767.s006.pdf]

```
Drosophila     1 ------------------------------------MASLYVGDLPQDVNESGLFDKFSSAGPVLSIRVCRDVITRRSLGYAYVNFQQPADAERALDTMN
Homo           1 ---------------------------MNAAASSYPMASLYVGDLHSDVTEAMLYEKFSPAGPVLSIRVCRDMITRRSLGYAYVNFQQPADAERALDTMN
C.elegans      1 ------MEMNVAAPAAAVAGAAAPQPGQNQTGSSYTMASLYVGDLHPDVNESILFEKFSAAGPVLSIRVCRDNATRLSLGYAYVNFQQPADAERAMDTMN
S.cervisiae    1 MADITDKTAEQLENLNIQDDQKQAATGSESQSVENSSASLYVGDLEPSVSEAHLYDIFSPIGSVSSIRVCRDAITKTSLGYAYVNFNDHEAGRKAIEQLN
P.berghei      1 ----------------------MIANSTNIMPPSFSTASLYVGDLSEDVTEAVLYEIFNTVGHVLSIRVCRDSVTRKSLGYAYVNYHNLADAERALDTLN

Drosophila    65 FDLVRNKPIRIMWSQRDPSLRRSGVGNVFIKNLDRAIDNKAIYDTFSAFGNILSCKVATDEKGNSKGYGFVHFETEEAANTSIDKVNGMLLNGKKVYVGK
Homo          74 FDVIKGKPIRIMWSQRDPSLRKSGVGNVFIKNLDKSIDNKALYDTFSAFGNILSCKVVCDENG-SKGYAFVHFETQEAADKAIEKMNGMLLNDRKVFVGR
C.elegans     95 FEALHGKPMRIMWSQRDPAMRRSGAGNIFIKNLDKVIDNKSIYDTFSLFGNILSCKVAIDEDGFSKGYGFVHFETEEAAQNAIQKVNGMLLAGKKVFVGK
S.cervisiae  101 YTPIKGRLCRIMWSQRDPSLRKKGSGNIFIKNLHPDIDNKALYDTFSVFGDILSSKIATDENGKSKGFGFVHFEEEGAAKEAIDALNGMLLNGQEIYVAP
P.berghei     79 YTNIKGQPARLMWSHRDPSLRKSGTGNIFVKNLDKTIDNKALFDTFSMFGNILSCKVATDEFGKSKNYGFVHYEDEESAKEAIEKVNGMQLGSKNVYVGH

Drosophila   165 FIPRKEREKELGEKAKLFTNVYVKNFTEDFDDEKLKEFFEPYGKITSYKVMSKEDGKSKGFGFVAFETTEAAEAAVQALNGKDMG---------------
Homo         173 FKSRKEREAELGAKAKEFTNVYIKNFGEEVDDESLKELFSQFGKTLSVKVMRDPNGKSKGFGFVSYEKHEDANKAVEEMNGKEIS---------------
C.elegans    195 FQPRAQRNRELGETAKQFTNVYVKNFGDHYNKETLEKLFAKFGNITSCEVMTVE-GKSKGFGFVAFANPEEAETAVQALHDSTIEG--------------
S.cervisiae  201 HLSRKERDSQLEETKAHYTNLYVKNINSETTDEQFQELFAKFGPIVSASLEKDADGKLKGFGFVNYEKHEDAVKAVEALNDSELN---------------
P.berghei    179 FIKKSER----ATNDTKFTNLYVKNFPDTVTEAHLKQLFSPYGEITSMIVKSD--NKNRKFCFINYSDADSARNAMENLNGKKITEDGKIDYNYDPKKEE

Drosophila   250 -------------------------------------------------------------------------------------------------EGK
Homo         258 --------------------------------------------------------------------------------------------------GK
C.elegans    280 -------------------------------------------------------------------------------------------------TDL
S.cervisiae  286 --------------------------------------------------------------------------------------------------GE
P.berghei    273 SEKAANENYNNNNTTSEENATTSETPAEKKTTDSESATNKDATPGEDQTSANGTTTTVTSTTDAKTEETPNDNTANAGINASITEKKDKKKSGESTETPN

Drosophila   253 SLYVARAQKKAERQQELKRKFEELKQKRHESVFGVNLYVKNLDDTIDDDRLRIAFSPYGNITSAKVMTDEEGRSKGFGFVCFNAASEATCAVTELNGRVV
Homo         260 IIFVGRAQKKVERQAELKRKFEQLKQERISRYQGVNLYIKNLDDTIDDEKLRKEFSPFGSITSAKVMLED-GRSKGFGFVCFSSPEEATKAVTEMNGRIV
C.elegans    283 KLHVCRAQKKSERHAELKKKHEQHKAERMQKYQGVNLYVKNLDETVDDDGLKKQFESYGNITSAKVMTDENGRSKGFGFVCFEKPEEATSAVTEMNSKMV
S.cervisiae  288 KLYVGRAQKKNERMHVLKKQYEAYRLEKMAKYQGVNLFVKNLDDSVDDEKLEEEFAPYGTITSAKVMRTENGKSKGFGFVCFSTPEEATKAITEKNQQIV
P.berghei    373 ILYVGPHQSRARRHAILKAKFDTLNTESRNKHPGVNLYIKNLDDSMNDQTLKELFEPYGTITSAKVMKDDKDQSKGFGFVCFGTHEEANKAVTEMHLKII

Drosophila   353 GSKPLYVALAQRKEERKAHLASQYMRHMTGMRMQQLGQIYQPN------------AASGFFVPTLPSNQR---FFGSQVATQMRNTPRWVPQVRPPAAIQ
Homo         359 GSKPLYVALAQRKEERKAHLTNQYMQRVAGMRALPANAILNQFQ----------PAAGGYFVPAVPQAQGRPPYYTPNQLAQMRPNPRWQQGGRP-----
C.elegans    383 CSKPLYVAIAQRKEDRRAQLASQYMQRLASMRMHGN------------------VPGAAMYNPTQPGPG----YYVANPMQQQRNFAGGQQMVRP-----
S.cervisiae  388 AGKPLYVAIAQRKDVRRSQLAQQIQARNQMR---------------------------------------------------------------------
P.berghei    473 NGKPLYVGLAEKREHRLSRLQQRFRMNPIRHHINNSLSSPIQYPNNQTPQLQFNQTLNYGRPVITTFNQNNLISWRHQQAAAQQQAVHQQAAAQQLGFN

Drosophila   438 GVQAGAAAAGGFQGTAGAVPTQFRSAAAGARGAQPQVQGTHAAAAAANNMRNTGARAITG----------------------------------------
Homo         444 ---QGFQGMPSAIRQSGPRPTLRHLAPTGSECPDRLAMDFGGAGAAQQGLTDS---CQSG----------------------------------------
C.elegans    456 ---GGRWGMQNQYPVQNQYMMAQGPGVYQNRMGRPQNQQGGPRGPPQQYNQVAQGGVRMQ----------------------------------------
S.cervisiae  419 ------------YQQATAAAAAAAAGMPGQFMPPMFYGVMPPRGVPFNGPNPQQMNPMGG----------------------------------------
P.berghei    573 AGLRGQINQMRLYTQNNMMNHNIGQNKASQQLHHNQQYSMGPNPQHQQTNLNAPGQANPQQLQGTAPVPNNQLLNNNMRNMNNRGNRNLPGMNMQSPKQM

Drosophila   498 ------------------------------QQTAAPNMQIPGAQIAGGAQQRTSNYKYTSNMRNPPVPQLHQTQPIPQQLQGKNSEKLIASLLANAKPQE
Homo         498 ------------------------------GVPTAVQNLAPRAAVAAAAPRAVAPYKYASSVRSP-HPAIQPLQAPQPAVHVQGQEPLTASMLAAAPPQE
C.elegans    513 ------------------------------GPPRTQNPGVQQQNVPRPPQQQQQQRPAPTGPKAPPQPYQAYQQRPQGIVIG-GQEPLTSAMLAAAAPQE
S.cervisiae  467 ------------------------------MPKNGMPPQFRNGPVYGVPPQ--------------------------------GGFPRNANDNNQFYQQK
P.berghei    673 PLNMIGAKQTNTQQNQPQGQPQGQPQGQPQAQPQVQSQQKTGQSIQQQPIPQNSNFKFTSQARNRMELPNKNANKNNMNPGYNNNTTLTAAALASAPPSM

Drosophila   568 QKQILGERLYPMIEHMHA--NLAGKITGMLLEIENSELLHMIEDQEALKAKVEEAVAVLQVHRVTEPAN-----------
Homo         567 QKQMLGERLFPLIQTMHS--NLAGKITGMLLEIDNSELLHMLESPESLRSKVDEAVAVLQAHHAKKEAAQKVGAVAAATS
C.elegans    582 QKQLLGERIYALIEKLYPGHKDAGKITGMMLEIDNSELIMMLQDSELFRSKVDEAASVLVSAQKQ---------------
S.cervisiae  505 QRQALGEQLYKKVSAKTSNEEAAGKITGMILDLPPQEVFPLLESDELFEQHYKEASAAYESFKKEQEQQTEQA-------
P.berghei    773 QKQVLGENLFPLVANYHP--TLAGKITGMMLEMDNSELLILLENEDQLKKKIDEALVVLQNAK-----------------
```
