# Supplementary material for: Universal Features of Post-Transcriptional Gene Regulation Are Critical for Plasmodium Zygote Development
Source: PLoS Pathog. 2010 Feb 12;6(2):e1000767. doi: 10.1371/journal.ppat.1000767 (PMC2820534; doi:10.1371/journal.ppat.1000767)
Supplement: Figure S6 — Homolog of Bruno (HoBo) PB001285.00.0. ClustalW alignment of Plasmodium berghei HoBo PB001285.00.0 (www.plasmodb.org) with homologs of Drosophila melanogaster (AAB58464.1 = BRUNO), human (BAD93011.1 = bruno-like 4 protein) and Caenorhabditis elegans (AAB37881.1 = Elav-type RNA binding protein family protein 1) recovered from BLASTP hits at www.ncbi.nlm.nih.gov. Identical and similar amino acids are indicated in black and grey shading, respectively. (0.03 MB PDF) [file ppat.1000767.s007.pdf]

```
Drosophila    1 --MFTSRASFLANRRMIFDFSEKNNDIYLDAFGMSDKSSSATNSLPNSPIHSNNNNPSLL
Homo          1 --------ARASERERGARECERERIKGKKIFSMYIKMATLAN-----------------
C.elegans     1 -------------MSGAVLPAVLVVPKMESVVVPIAESTVIN-----------------
P.berghei     1 MNNNTQQQQQQQQQQHQQQQPQQPQQQQQQQQQQQQQQQQQQYS-----------------

Drosophila   59 NNNNNNSNGTSSNNSLNVGNNNSNPSLGGTNSNALVSVGSNGIMSAAGLVNNNNNPCSAN
Homo         36 --------GQADNASLSTNGLGSSPGSAGHMN---------------GLSHSPGNPSTIP
C.elegans    30 ---------AKDTTPSTAADNDVSPSPS--------------------------------
P.berghei    44 --------NENNESSYGEARINEQPQYIYQMNN---------------------------

Drosophila  119 RNVVAMVDDDACFRLDTDATVTYGEKEPDPDNIKMFVGQVPKSMDESQLREMFEEYGAVH
Homo         73 --------------------------MKDHDAIKLFIGQIPRNLDEKDLKPLFEEFGKIY
C.elegans    49 --------------------------EPDTDAIKMFVGQIPRQWNETDCRRLFEKYGSVF
P.berghei    69 -----------------------PYNPAPSIPVKLFVSSIPKNLTEDDIKLIFEEYGATK

Drosophila  179 SINVLRDKATGISKGCCFVTFYTRRAALKAQDALHNVKTLNGMYHPIQMKPADSENRN--
Homo        107 ELTVLKDRFTGMHKGCAFLTYCERESALKAQSALHEQKTLPGMNRPIQVKPADSESRGGS
C.elegans    83 SCNILRDKSTQASKGCCFVTFYHRKDAIEAQGALHNIKVIEGMHHPVQMKPADTENRN--
P.berghei   106 DVVFIKDKKPNANRANVFVRMESIYFAQKAIEDLHGKKIICESLGPLIVKFAIGELEKYG

Drosophila  237 ---------ERKLFVGMLNKKLNENDVRKLFEVHGAIEECTVLRDQNGQSKGCAFVTFAT
Homo        167 SCLRQPPSQDRKLFVGMLNKQQSEDDVRRLFEAFGNIEECTILRGPDGNSKGCAFVKYSS
C.elegans   141 ---------ERKLFIGQLSKKHNEENLREIFAKFGHIEDCSVLRDQDGKSRGCAFVTFTN
P.berghei   166 INMNNANENEAKLFVGSLPKDITDDQIRNIFNRYGNVKEVYIMKNSNGVSKRCAFVNYDY

Drosophila  288 KHAAISAIKVTLSQNKIMEGCTSPLVVKFADTQKEKEQKKIQQIQAN-------------
Homo        227 HAEAQAAINALHG-SQTMPGASSSLVVKFADTDKERTMRRMQQMAGQ-------------
C.elegans   192 RSCAVVATKEMHH-SQTMEGCSAPLVVKFADTQKDKDVKTKSMITGSGAGSPKGGAASLL
P.berghei   226 KEQGIFAVQNLNG-KIAIENAEKPIEVRFAQSKNQLQER---------------------

Drosophila  335 ----------------LWNLASNINIPLGQTATSVTTPILP-------------------
Homo        273 ----------------MG-MFNPMAIPFGAYG-AYAQAMQQ-------------------
C.elegans   251 QNLNPALLQQLGGGQNYQAVASLLSLINGQQGQQQQHQQQQNVLGILGTVLSALGKLTEG
P.berghei   264 ------------------------------------------------------------

Drosophila  360 --------------NPPQQPSPVLGADAITPASIQLLQQLQAVGLQHQLLQALTGLGAQQ
Homo        296 --------------QAALMASVAQGGYLNPMAAFAAAQMQQMAALNMNGLAAAPMTPTSG
C.elegans   311 GDDASAKSSSEKPRHQALMTSPAPTATSSTSSSASHHHQQHQQQLSQQQQQQQHPQQQGL
P.berghei   264 --------------------------QLLNRVLMNPLQMNNNNIDDNNNNGNTINNNSNN

Drosophila  406 SSSATDTSAVAAAGLLTPMTVQNLAAIAAMTQPS-----------------------LSN
Homo        342 GSTPPGITAPAVPSIPSPIGVNGFTGLPPQAN-G-----------------------QPA
C.elegans   371 GNPLLGNPAMAAQNQFDAITMAQIAHQQQMLALQGFAVQQGAPSQQQQGLAGGMAGAKTT
P.berghei   298 NSNKSNNNAYMKSKHFKNMNINTFNRYPMHMNYN-------------------------L

Drosophila  443 AAAAAARATSPGSAQLTNTAALLWSDPNPMASAYMSTAAGLPQFGSASALSTSPLASVAL
Homo        378 AEAVFANGIHPYPAQSP-TAADPLQQAYAGVQQYAGPAAYPAAYGQISQAFPQPPPMIPQ
C.elegans   431 SPVAASLANHQQIALTPFAGGAAALDHFQAMQQYALLANLQATGGVGVQATTSAAQMVGN
P.berghei   333 QNNSNQQRNNNNSIRSPWKQYFSKDDGRPYYHNELTGQTQWHKPRKMDQDFINPLNMN--

Drosophila  503 SAAAAAAAGKQIEGPEGCNLFIYHLPQEFTDTDLASTFLPFGNVISAKVFIDKQTSLSKC
Homo        437 ---------QQREGPEGCNLFIYHLPQEFGDAELMQMFLPFG------------------
C.elegans   491 ---------GDVKGPDGANLFIYHLPQDFGDSDLINTFAPFGGILSAKVFIDKVTNLSKC
P.berghei   391 -----------EVGPVGANIFIFHIPNEWIQNDLLAAFSPFGNIISAYIATEKDTGRNRG

Drosophila  563 FGFVSFDNPDSAQVAIKAMNGFQVGTKRLKVQLKKPKDS-KPY-----------------
Homo        470 --FVSFDNPASAQTAIQAMNGFQIGMKRLKVQLKRPKDANRPY-----------------
C.elegans   542 FGFVSYENAQSATNAISAMNGFQIGSKRLKVQLKVDRGNPYNR-----------------
P.berghei   440 FAFVSYDNVDSAINAVKYMNGFLAHKKKLKVTIKKGEEQYVQALINQRNQLNNTDNRRNF
```
