# Supplementary material for: Universal Features of Post-Transcriptional Gene Regulation Are Critical for Plasmodium Zygote Development
Source: PLoS Pathog. 2010 Feb 12;6(2):e1000767. doi: 10.1371/journal.ppat.1000767 (PMC2820534; doi:10.1371/journal.ppat.1000767)
Supplement: Figure S7 — Homolog of Musashi (HoMu) PB000805.02.0. ClustalW alignment of Plasmodium berghei Musashi-like PB000805.02.0 (www.plasmodb.org) with homologs of Drosophila melanogaster (musashi; NP_733108.2), human (hnRNP A2/B1; EAW93834.1), Caenorhabditis elegans (musashi; NP_497799.1) and Arabidopsis thaliana (UBA2A; NP_567042.1) recovered from BLASTP hits at www.ncbi.nlm.nih.gov. Identical and similar amino acids are indicated in black and grey shading, respectively. (0.03 MB PDF) [file ppat.1000767.s008.pdf]

```
Drosophila musashi    1 ----MLFENPAVAAKLPFPYNVPPPLQAAAAAAAAVPNLRFQTPIKAFAC
C.elegans MuSashI     1 --------------------------------------------------
human hnRNP A2/B1     1 --------------------------------------------------
Arabidopsis UBA2A     1 MTKKRKLEGEESNEAEEPSQKLKQTPEEEQQLVIKNQDNQGDVEEVEYEE
PB000805.02.0         1 --------------------------------------------MEEKNLS

Drosophila musashi   47 LTAATRSVSEMNATSLYAGNPMENAAAAAAAAAAGLIDPHHNRDLHQALV
C.elegans MuSashI     1 ---------------------MTTTVSTGATAVATLRETSPPVDGHEEAR
human hnRNP A2/B1     1 --------------------------------------------------
Arabidopsis UBA2A    51 VEEEQEEEVEDDDDEDDGDENEDQTDGNRIEAAATSGSGNQEDDDDEPIQ
PB000805.02.0         8 VNNTNINDPNQCDTSSNLNASQDNNDNILENQNLNDKKIKDEESEVEQLR

Drosophila musashi   97 ASIANNSVAAIGGGLTTAAVLKSAAQQSQQAVQQNQNAVVVTPGLEQPKQ
C.elegans MuSashI    30 LNADS---------------------------------------------
human hnRNP A2/B1     1 --------------------------------------------------
Arabidopsis UBA2A   101 DLLEPFSKEQVLSLLKEAAEKHVDVAN-----------------------
PB000805.02.0        58 RLIAPLSKEQLIDILATAASIHEDIRDR----------------------

Drosophila musashi  147 EPAQQAALALLKENVNASAGAGQNNGQAAMGGSNKSGSSGRSTPSLSGGS
C.elegans MuSashI    35 ----------------------------------------------DDGS
human hnRNP A2/B1     1 ------------------------------------------------ME
Arabidopsis UBA2A   128 --------------------------------------------RIREVA
PB000805.02.0        86 ---------------------------------------------CNDAV

Drosophila musashi  197 GSDPAPGKLFVGGLSWQTSSDKLKEYFNMFGTVTDVLIMKDPVTQRSRGF
C.elegans MuSashI    39 HGSQDPGKMFIGGLSWQTTAENLRDYFGRFGEVNECMVMRDPATKRARGF
human hnRNP A2/B1     3 REKEQFRKLFIGGLSFETTEESLRNYYEQWGKLTDCVVMRDPASKRSRGF
Arabidopsis UBA2A   134 DEDPVHRKIFVHGLGWDTKTETLIEAFKQYGEIEDCKAVFDKISGKSKGY
PB000805.02.0        91 TSSPSTRRLMVRNIPFSTKDEQFLKYFETFGEIEDGIIVREKE-GRSKGY

Drosophila musashi  247 GFITFQEPCTVEKVLKVPIHTLDGKKIDPKHATPK-N-------------
C.elegans MuSashI    89 GFITFVDPSSVDKVLNNREHELDGKKIDPKVAFPKRT-------------
human hnRNP A2/B1    53 GFVTFSSMAEVDAAMAARPHSIDGRVVEPKRAVAREESG-----------
Arabidopsis UBA2A   184 GFILYKSRSGARNALKQPQKKIGSRMTACQLASKGPVFGGAPIAAAAVSA
PB000805.02.0       140 GFVTFKYIESVQKCLKG-SHTLDNKDLQVRLVAD----------------

Drosophila musashi  283 ---RPRQANKTKKIFVGGVSQDTSAEEVKAYFSQFGPVEETVMLMDQQTK
C.elegans MuSashI   126 ---QAKLVTKTKKVFIGGLSATSTLEDMKQYFETYGKVEDAMLMFDKATQ
human hnRNP A2/B1    92 ---KPGAHVTVKKLFVGGIKEDTEEHHLRDYFEEYGKIDTIEIITDRQSG
Arabidopsis UBA2A   234 PAQHSNSEHTQKKIYVSNVGAELDPQKLLMFFSKFGEIEEGPLGLDKYTG
PB000805.02.0       173 ----PFTDHYQNKLFVRNLSQKTNVTTLRNIFEKYGKLEECVIIHDNE-G

Drosophila musashi  330 RHRGFGFVTFENEDVVDRVCEIHFHTIKNKKVECKKAQ----PKEAVTPA
C.elegans MuSashI   173 RHRGFGFVTFDSDEVADKVCEIHFHEINGKMVECKKAQ----PKEVMLPV
human hnRNP A2/B1   139 KKRGFGFVTFDDHDPVDKIVLQKYHTINGHNAEVRKAL----SRQEMQEV
Arabidopsis UBA2A   284 RPKGFCLFVYKSSESAKRALEEPHKTFEGHILHCQKAIDGPKPGKQQQHH
PB000805.02.0       218 KSKGYGFLTFSSPKEAFKVMQQPERIIDNRVVFLHFAVS-----QNYKKY

Drosophila musashi  376 AQLLQKRIMLGTLGVQLPTAPGQLIGARGAGVATMNPLAMLQNPTQLLQS
C.elegans MuSashI   219 QLNKSR----AAAARNLYGMPPETLLAYAQYLPRFGGNLMYPNFTNVFNN
human hnRNP A2/B1   185 QSSRSGRGGNFGFGDSRGGGGNFGPGPGSNFRGGSDGYGSGRGFGDGYNG
Arabidopsis UBA2A   334 HNPHAYNNPRYQRNDNNGYGEPGGHGHLMAGNPAGMGGPTAGVINPATGQ
PB000805.02.0       263 QNNHAYIKKNPNFNYYQKNNPNNRNNFIRRTPVYYRENESPNTFNYPVSF

Drosophila musashi  426 PAAAAAAQQAALISQNPFQVQNAAAAASIANQAGFGKLLTTYPQTALHSV
C.elegans MuSashI   265 MPGGYSGLSTPGGSSNRPPHQFDTASLYSLNNG--GQLLDSQAQMFMN--
human hnRNP A2/B1   235 YGGGPGGNYGSGNYNDFGNYNQQPSNYGPMKSGNFGGSRNMGGPYGGGNY
Arabidopsis UBA2A   384 ALTALLASQGAGLAFNPAIGQALLGSLGTAAGVNPGNGVGMPTGYGTQAM
PB000805.02.0       313 VPNVYSNIPHGYQFNYPGNLDSFNAFYNNPRY------------------

Drosophila musashi  476 RYAPYSIPASAATANAALMQAHQAQSVAAAAHHHQQQQQQQHHHQQQTHN
C.elegans MuSashI   311 -------------------------------------QQSYHSHSKY---
human hnRNP A2/B1   285 GPGGSG------------------------------GSGGYGGRSRY---
Arabidopsis UBA2A   434 APGTMP------------------------------GYGTQPGLQGGYQT
PB000805.02.0           --------------------------------------------------
```
