# Supplementary material for: Universal Features of Post-Transcriptional Gene Regulation Are Critical for Plasmodium Zygote Development
Source: PLoS Pathog. 2010 Feb 12;6(2):e1000767. doi: 10.1371/journal.ppat.1000767 (PMC2820534; doi:10.1371/journal.ppat.1000767)
Supplement: Figure S8 — The ALBA domain proteins of Plasmodium berghei and selected Alveolata. (A) Partial sequence alignment (the highly divergent C-termini of the proteins shown have been omitted) of members belonging to the MDP2-like group. (B) Complete sequence alignment of members of the Rpp20-like group. Proteins were aligned using ClustalW at www.ch.embnet.org using default setting. The grey bar indicates the position of the Alba domain (PF01918) according to www.pfam.org. All protein identifiers (e.g. Q4Z4H5_PLABE) are from www.uniprot.org and indicate the following species: PLABE Plasmodium berghei, PLAF7 Plasmodium falciparum, TOXGO Toxoplasma gondii, 9ALVE Perkinsus marinus, BABBO Babesia bovis, THEPA Theileria parvum, 9CRYT Cryptosporidium muris. (0.03 MB PDF) [file ppat.1000767.s009.pdf]

A

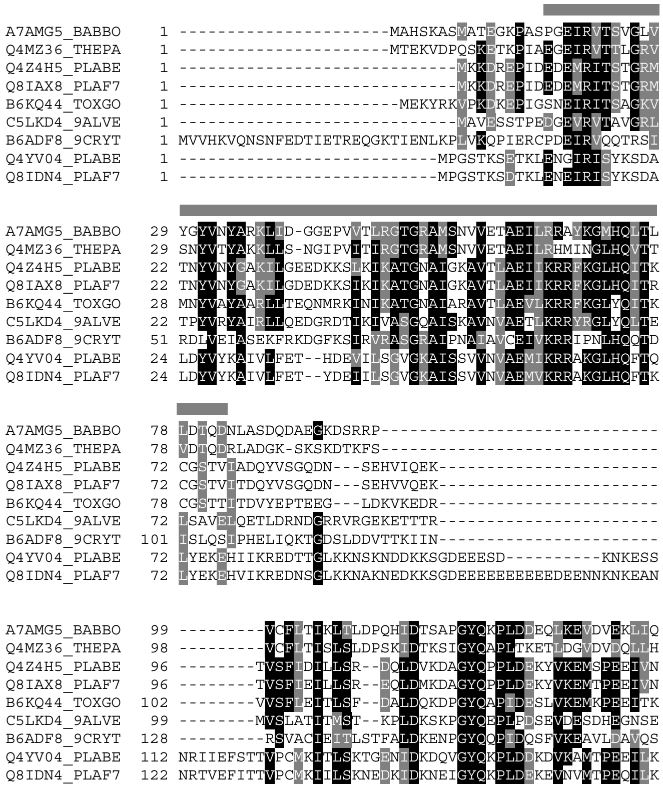

Q4Z4H5_PLABE = PB000862.00.0 (Alba-1)
Q4YV04_PLABE = PB000812.02.0 (Alba-2)

B

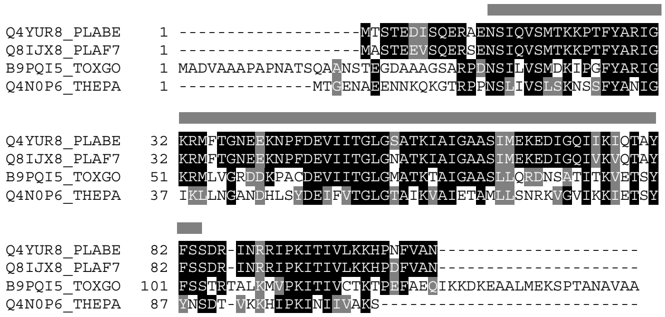

Q4YUR8_PLABE = PB000878.02.0 (Alba-3)
