# Supplementary material for: Universal Features of Post-Transcriptional Gene Regulation Are Critical for Plasmodium Zygote Development
Source: PLoS Pathog. 2010 Feb 12;6(2):e1000767. doi: 10.1371/journal.ppat.1000767 (PMC2820534; doi:10.1371/journal.ppat.1000767)
Supplement: Figure S9 — Partial ClustalW alignment of Plasmodium berghei PGAM PB001107.03.0 (www.plasmodb.org) with orthologs of P. chabaudi (PC001355.02.), P. falciparum (PFC0430w), P. knowlesi (PKH_082990), P. vivax (PVX_119620) and P. yoelii (PY07389) recovered from BLASTP hits at www.plasmodb.org. Identical and similar amino acids are indicated in black and grey shading, respectively. (0.05 MB PDF) [file ppat.1000767.s010.pdf]

```
PB001107.03.0  137 TENNS-------------------------------------------------TKINELINNIYYKISYYLKKNRKYLNKILQQNYEINIYLIRHMEAQ
PC001355.02.0    1 ----------------------------------------------------------------------------------------------------
PFC0430w       130 -------------------------------------------------------KHNDMIGDVYYKIAYYLKKNRKYMQRILKKNYVIKIYLVRHMEAL
PKH_082990     268 NEGAS--------NYAHHSYGTPESAQTPNYEFGGAKYHHYDPPHLPHKGNESHVKFNELISDVYYKSSYYLKRNRKYMQNLLKSNCVINIYLVRHMEAM
PVX_119620     299 NHSASNYASNYASNYGYHSYGAQESGHQASYEHGGAKYHHYHHPHK---GNEVHVKYNELISDVYYKSSYYLKKNRKYMQKLLKTNCVINIFLVRHMEAD
PY07389        136 NENNS-------------------------------------------------TKINELINNIYYKTSYYLKKNRKYLNKILQQNYEINIYLVRHMEAQ

PB001107.03.0  188 HNKQHVEDR-YPRDLIYKRPIYLDSVATEEGIKMCEDVRNYHLNGTGKSGCNKMHDIYYKEIIQLYNDYIEKCDKEQQTSKEN----SKESRKESSK---
PC001355.02.0    1 ----------------------------------------------------------------------------------------------------
PFC0430w       175 HNTEVIKNSTISRDMIYRNKDFFDCPASEEGKIKCEQLK-------------SKDNKLYQKLIKLYNESVHGKDEQTGDINTNHQKYNTKNINNNHNNNN
PKH_082990     360 HNREVLDKTDKVRDLIYKDIKYLDCGASEQGKQMCENLK------------KDGNNRLYQKVMELYNESLKKVDIPSTSGVDISGATNKGSVTPPSN---
PVX_119620     396 HNREVLESKDKARDLIYKEIKYLDCKASEQGKQMCEDLK------------NDANNRMYQKIMQLYNDSLQRMDVPPVGGADRSGASNKGATIKGASNPG
PY07389        187 HNKKHVEDLDTPRDLIYKQPIYLDSVATEDGIKMCETVRNYHLNGTGKSGCNQMHDIYYKEIIQLYNDYVEKCDKEQQISKENGKENSKESGKESGK---

PB001107.03.0  280 ---------------------------------------------------------------------------ENKKENKKGVFERNNNFVIISSPLR
PC001355.02.0    1 -------------------------------------------------------------------------------------FERNNDFVIICSPLR
PFC0430w       262 NNNNNNNHNNNNHNNNNHNNNNHNNNNHNNNNHNIYSN---------------------------SLHNNQSYNNLRINEHAKGAFEKNNNFVIITSPLR
PKH_082990     445 ----------------------------------------------------------------------STPPVDPVGSAKKGILERNNDFIIISSPLR
PVX_119620     484 ASNQGASNQGASNQGASNQGASNQGASNQGASNQGASNQGDSNQGASNPGASNPGASNQGASNQGASNQPSNSAANPGATPRKGILERNSDFVIISSPLR
PY07389        284 ---------------------------------------------------------------------------EGRKENKKGVFERNNNFVIISSPLR

PB001107.03.0  305 RCLQTMKYLFNFKKNIVIYEPIREISGSYITDQRSKTSEVKKFCDNNFDEYELMCFGEEDIMSVERFRESSCQVYFRCLQFLKFVHSLAINYFASIEREN
PC001355.02.0   16 RCLQTMKYLFNFKKNIVIYEPIREISGNYVSDQRSKTSEVKKFCDNNFDEYELMCFGEEDIMNVERCRESACQVYFRCLQFLRFAHSLAINYFASIEREN
PFC0430w       335 RCLETTKYFLNFKKNIIVYEAVRETAGNYYSDQRSKTSDIKKFCDQNFEDYELICFGETDALSGNKFRETSEQVYCRCLQFLKLIHALAINYFASLEKIN
PKH_082990     475 RCLETMKYYFNFKKNILIYESVREMAGNYSSDQRSKTSDVKKFCEQNFEDYEVVCFGENDIFYGDKFRESSDQVYCRCLQFLKMAHSLAVNYFASLEREN
PVX_119620     584 RCLETMKYYFNFKRNILVYESVREMAGNYFSDQRSKTSEVKKFCESNFEDYEVICFGENDILFGEKFRESSDQVYCRCLQFLKMAHALAVNYFASLERDS
PY07389        309 RCLQTMKYLFNFKKNIVIYEPVREISGSYITDQRSKTSEVKKFCDNNFDEYELMCFGEEDIMHVERFRESSCQVYFRCLQFLKFVHSLAINYFASIEREN

PB001107.03.0  405 DSMSLDVKIGNSKSPKGNSNQSNMKNNE---------EKQNSTPNSEIDDYINDKCNNEETCTKTN----------------------------------
PC001355.02.0  116 DSMGLDGKASNSKSPQGSNNQSNLKNDE----------KKNSTQNSEIGDYMSDKCNNEDTCAKTN----------------------------------
PFC0430w       435 EQEEINNKNFENINSQTCTNDIDENVENEKLCSASQNDNSFENKGNKVINNLNNSGKVNEKKKKKAEMKDDNEKKEFDENNIKEEEEEEEYDNDDDFTVE
PKH_082990     575 GEEDDAEEADKREDGTEENQMPGGGNVEKDLCGGMGGPTGEVMEKSCEEGKIKREDHVQQECQENLTGEGLVAEVSTCKKSEQEGKVVDGG----EVADG
PVX_119620     684 -------EEAAGEAGEDLCGAVDGAAGEVAAAEGVDKSVEEVQVKREVEVEPEGEAAPEVEVPRDTQEGNPLEKESQCGKSGQGGDAAEG-----DAPNG
PY07389        409 DSMSLDVKVGNTNQSSMKNNEEKQNSAQNSAQNSAQNSAQNSAQNSEIDDHINDKCNNEEICTKKN----------------------------------

PB001107.03.0  462 ------------------EKNKKSFNIVVVSHSSYLLHLLALLDYLNLDARNFNNCDIRKITIPLTNTFLFFNNIINLQLAKPVISNNMPLCFRDKQKNV
PC001355.02.0  172 ------------------GKNKKVFNLVVVSHSSYLLHLLALLDYLNLDAR-FNNCDIRKITIPLTNTFLFFNNIINLQLAKPVISNNMPLCFRDKQKNV
PFC0430w       535 NNKYVDDEKMNDEMVKKPKNKKGVYNVVMVSHSSFIVHMLALLDYLDLDFRGLNNCDIRKISIPLSHSFLFYNNITNLQIARPLSINNIPISYMNKNK-A
PKH_082990     671 GKVSDEDKNLANEKKSKGKEKKRVYNIVLVSHSSFIMHFLALLDYFDLGARNFNNCEIRKVVIPLSNTFLFFNNVVNMQLAKPVCNNNFPIVFRNKNK-I
PVX_119620     772 VDTAEVQKNPANENQPQESEKERVFNIVLVSHSSFISHFLALLDYFNLDERNFNNCEIRKVTLPLSNTFLFFNNVVNMQLARPLCTNNFPLVFRNKNK-V
PY07389        475 ------------------GNNKKVFNIVVVSHSSYLLHLLALLDYLNLDDRNFNNCDIRKITIPLTNTFLFFNNIINLQLAKPVISNNMPLCFRDKQK--

PB001107.03.0  544 LKKTYKDKNIYTLNSINDIDDIICESPCTIIIYQYNELIKNQNNYKKYLEKIKNFIDNNNKEFQ---LKNGYFSNGMYKKYVLKKGKNDDNS---ENNMY
PC001355.02.0  253 LKKTYKDKNIYTLNNINDLDDIICESPCTIMIYQYHEVIKDQNNYKKYLEKIQNFIDNNNKEFQ---SKKGYFSNGMYKKYVLKKGKNEDS-----NDMY
PFC0430w       634 LKKAYKDKYIYTINSYTELDDLICSQPCTVIIYQYDMLIKNEK-NKKYLDSVQKFIDNSNNYYSNSNKENHVYSNGLYKNYVLRNKR-------------
PKH_082990     770 LKKAFKDKNIYTLSTYNDFDELTCQHPCTVVIYQYASLVNEEK-YMKYLEKVKNFIDLANKEHE---QEEKFFSNGMFKNYVKEDKDN------------
PVX_119620     871 LKKAFKDKNIYTLSTYNDFDEMTCQQPCTLVIYQYAALVKEEK-YQKYLEKVKSFVQLANKEHE---EEEQIYSNGMFKNYVKKEKKE------------
PY07389        555 ---------------------------------NYNELIKNQNNYKNYLEKIKNFIDNNNKEFQ---LKNGYFSNGMYKKYVLKKGKNDDNNNNGENNMH

PB001107.03.0  638 NLGRDILVTDASEHLGFSERKQAWELNRKGYIAGQNVILIVLPDVENAPKKTNAPNNKGIETDLDTKTNDDDN----EDLGSLKNVTKIIDNYDSVQDLI
PC001355.02.0  345 NLGRDILVTDASEHLSFSERKQVWELNKKGFIAGQNIILIVLPDVDSVPKKANAPMNKSIEMHLDTKINENNN----EDLGSLKNVTKIVDNYDSVQDLI
PFC0430w       720 -LGRNVLIYDGSEYLIYVPEKGSWENNKKGYIVGQNIILVVLPEVESKIINKENVGEEEYVSLNTKNKEQNEN--YKKTSHYLQNVIEVIKNYDSIQDLI
PKH_082990     854 -MGRSVLVSDASEHILYVKEKNAWEVNKKGFFVGQNVVIIVLPHVEPTVSSNNVNISNNTKEKPTEKSAHLDN------------VLQVVNNYNHVQDLI
PVX_119620     955 -MGRSILVTDASEHLLYVPKKNAWEVNRKGFFVGQNVVIIVLPDVEPTASANNPSKSKDPKDKSNELSLHMDS------------VIQVVNNYKHVQDLI
PY07389        619 NLGRDILITDASEHLSFSERKQAWELNKKGYIAGQNVILIVLPDVENAPKKTNAPNNKGNETDIDTKINDDDDNNDKEDLGSLKNVTKIIDNYDSVQDLI

PB001107.03.0  734 KSPDFWISIKKNIDNINLNSFHEYIIKKYNKILTTENED-FCYIDLVASLENKNFINKYGNMFNGYFKKLKEKYNNNNSSLNLEKECKYINENLINKIFP
PC001355.02.0  441 KSTDFWISIKQNIHNINLNSFHEYIIKKYNKMLMKEDED-YCYIDLVASLENKNFINKYGSMFNGYFKKLKEKYNNNHLSLDLEKECKHINENLVSKMFP
PFC0430w       817 KSIDFWITLKENIKNIDIPTFQKYLIEKYNKIIKN--EMDVCYVDIVGCIENEEFMKKNEHKCNGYLKSLKEKYEKNND-INFEEECKYINENFIEEIFT
PKH_082990     941 KSKDFWKVVKENINNMDLNTFQEYLIGKYTKNVKSEEADNVCYLDLSSCLESKSFMDTFQDRCNGYLKRLKERCD-AFPYVDLENECRYINENLLMDMFP
PVX_119620    1042 KSKDFWKVLKDSISHLDLNTFQEYLIGRYTKVVTSEEADSFCYLDLASCLESKTFMEKFQDRCNGYLKRLKERCD-AFAYVDLEQECRHINENLLTEMFP
PY07389        719 KSPDFWISIKKNIHNINLNSFHEYIIKKYNKILMTENED-FCYIDLVASLENKNFINKYGNMFNGYFKKLKEKYNNNNSLLDLEKECKYINENLINKIFP

PB001107.03.0  833 QEYETSKDEDETGEVSMNSNLTSQRNTLNNHDKINIVKKKFIYIPNKYNDKNKMTKQ------KNNDNYNISNSIVPN------------NKAFVMENIK
PC001355.02.0  540 QDYEMSKDGNETGKTSTN---------SNSHDKINIVKKKFIYIPKKCNDKSRMTNQ------KNNDTYNISNNIIPN------------NKTFVMENIK
PFC0430w       914 SDYSSPNLDIKNEKDISSDNKNKNKNNDNNNNNNNNNNDDDFHNNNSYIQNNDFLEENIIRKKFIYIPKNDSNNIKIQSNIKQTYKDEKQNKSFLQENIN
PKH_082990    1040 REYASGTRGRLAAGSGNRSGDWTNGKEDTTLDQNTLVRKKFMCIPRRAAASAKVAAE------ALFGHSNKSTTVAVT---SGGGDSPVSSKNFLQENIN
PVX_119620    1141 REYPSSGVSGRLPG----TGDRATGKEDALLDERTLVRKKFMCIPRRAAVTAKVAAQ------ALFGHSNQAATLGAASLAAGGGEALATSKNFLQENIN
PY07389        818 HEYETSKDENENGKVLTN--LTSPKNTLNNHDKINIVKKKFIYIPNKYNDKNKMTKQ------KNNDSYNISNNIVPN------------NKTFVMENIK

PB001107.03.0  915 VLKSFPQSIQNLNVEMFYRDKFFYFNCLHKFIKSKNKIEGLVLAKLLAFLDLLKAFNFNSTSKIFQKLTKLDDIIYTNTIEDSS-QIVNTYSCDKKPVPV
PC001355.02.0  613 VLKSFPQSIQNLNVEMFYRDKFFYFNCLHKFIKSKNKIEGLVLAKLLAFLDLLKSFNFNSTNKIFQKLIKLNDLIDTNTIEDSS-QIVNTYSCDKTGVPV
PFC0430w      1014 ILKSFPQSIQNLSVEMFYRDNYFYFHCLNKYIKSKNKIEGLIICKLLTYLDLLRSYNTHGLNKTFQRLMHIEEKIDNNTKGIG--ETLNTYSCDINPVLA
PKH_082990    1131 TLKSFPQSIQNLSVEMFYRDHFFYFHCLHRFIKSKNKVEGLIISKLLTFLDLLRAFDVSATNKIFQKLSKLEGLIENNTCLDEEDRLVNTYSCDKKCAQV
PVX_119620    1231 TLKSFPQSIQNLNVEMFYRDHFFYFHCLHRFIKSKNKVEGLIISKLLAFLDLLRAFDVSATNKIFQKLSKLEGLIENNTSLDEEDRLVNTYSCDKKCAQA
PY07389        898 VLKSFPQSIQNLNVEMFYRDKFFYFNCLHKFIKSKNKIEGLVLAKLLAFLDLLKSFNFNSTNKIFQKLTKLDDVIYTNTIEDSS-QIVNTYSCDKKPAPV

PB001107.03.0 1014 NNTFYFSASAKKYKDIQTLKGMPYLYTPHYLLYFIFIFF---------------
PC001355.02.0  712 NNTLYFSAAAKKYKDIQALKG---------------------------------
PFC0430w      1112 NNTSYYGEGPKKYKIIYSIPGKKIKKNKIKKNKIK-------------------
PKH_082990    1231 NNTSYYDESARKYKLLQTLRGRKKKVVDDLNYMRYNLLTVAGGAENVFILNVLK
PVX_119620    1331 NNTSYYDESARKYKAIQTLRGRKKGVVDDLNYMRYNLLTVSGGAENVFILNVLK
PY07389        997 NNTFYFSASAKKYKDIQALKGRKRGIVDDLNYIRYNMLTISAGAENVYILNVLS
```
