# Supplementary material for: Universal Features of Post-Transcriptional Gene Regulation Are Critical for Plasmodium Zygote Development
Source: PLoS Pathog. 2010 Feb 12;6(2):e1000767. doi: 10.1371/journal.ppat.1000767 (PMC2820534; doi:10.1371/journal.ppat.1000767)
Supplement: Figure S10 — Enolase PB000456.03.0. ClustalW alignment of Plasmodium berghei Enolase PB000456.03.0 (www.plasmodb.org) with homologs of Drosophila melanogaster (P15007), human (NP_001966.1), Caenorhabditis elegans (NP_001022349.1) and yeast (P00924) recovered from BLASTP hits at www.ncbi.nlm.nih.gov. Identical and similar amino acids are indicated in black and grey shading, respectively. (0.03 MB PDF) [file ppat.1000767.s011.pdf]

```
C.elegans      1 ------------------------------------MFVPFTSAAHSLTSLIRGGGQPSK
S.cervisiae    1 ------------------------------------------------------------
Drosophila     1 MSWTASVFLRTSTTSMKFLRLRWPLPRIPQNKSANVAPRFRSKSAVSQLSSGFKFVQIRK
Homo           1 ------------------------------------------------------------
P.berghei      1 ------------------------------------------------------------

C.elegans     25 SNLSGQRMPITKIHARQIYDSRGNPTVEVDLFTEKGVFRAAVPSGASTGVHEALELRDGD
S.cervisiae    1 -------MAVSKVYARSVYDSRGNPTVEVELTTEKGVFRSIVPSGASTGVHEALEMRDGD
Drosophila    61 STCDSNEMTIKAIKARQIYDSRGNPTVEVDLTTELGLFRAAVPSGASTGVHEALELRDND
Homo           1 -------MSIEKIWAREILDSRGNPTVEVDLYTAKGLFRAAVPSGASTGIYEALELRDGD
P.berghei      1 -----MAHVITRINAREILDSRGNPTVEVDLETTLGIFRAAVPSGASTGIYEALELRDND

C.elegans     85 KAVHLGKGVLKAVSNINEKIAPALIAKGFDVTAQKDIDDFMMALDGSE------NKGNLG
S.cervisiae   54 KSKWMGKGVLHAVKNVNDVIAPAFVKANIDVKDQKAVDDFLISLDGTA------NKSKLG
Drosophila   121 KANYHGKSVLKAVGHVNDTLGPELIKANLDVVDQASIDNFMIKLDGTE------NKSKFG
Homo          54 KQRYLGKGVLKAVDHINSTIAPALISSGLSVVEQEKLDNLMLELDGTE------NKSKFG
P.berghei     56 KSRYLGKGVQQAIKNINEIIAPKLIG--LDCREQKKIDNMMVQELDGSKTEWGWSKSKLG

C.elegans    139 ANAILGVSLAVAKAGAVHKGLPLYKYIAELAG--TGKVVLPVPAFNVINGGSHAGNKLAM
S.cervisiae  108 ANAILGVSLAASRAAAAEKNVPLYKHLADLSKSKTSPYVLPVPFLNVLNGGSHAGGALAL
Drosophila   175 ANAILGVSLAVAKAGAAKKGVPLYKHIADLAG--NKEIILPVPAFNVINGGSHAGNKLAM
Homo         108 ANAILGVSLAVCKAGAAERELPLYRHIAQLAG--NSDLILPVPAFNVINGGSHAGNKLAM
P.berghei    114 ANAILAISMAICRAGAAANKTSLYKYLAQLAGKNTEKMILPVPCLNVINGGSHAGNKLSF

C.elegans    197 QEFMILPVGASSFAEAMRMGSEVYHHLKAEIKKRYGLDATAVGDEGGFAPNIQDNKEGLD
S.cervisiae  168 QEFMIAPTGAKTFAEALRIGSEVYHNLKSLTKKRYGASAGNVGDEGGVAPNIQTAEEALD
Drosophila   233 QEFMILPTGATSFTEAMKMGSEVYHHLKNVIKAKFGLDATAVGDEGGFAPNIQSNKEALN
Homo         166 QEFMILPVGAESFRDAMRLGAEVYHTLKGVIKDKYGKDATNVGDEGGFAPNILENSEALE
P.berghei    174 QEFMIVPVGAPSFKEAMRYGAEVYHTLKSEIKKKYGIDATNVGDEGGFAPNILNAHEALD

C.elegans    257 LLNTAIDKAGYTGKISIGMDVAASEFFKDG--KYDLDFKNPASDSSKWLSGEQLTELYQS
S.cervisiae  228 LIVDAIKAAGHDGKVKIGLDCASSEFFKDG--KYDLDFKNPNSDKSKWLTGPQLADLYHS
Drosophila   293 LISDAIAKAGYTGKIEIGMDVAASEFYKDG--QYDLDFKNEKSDKSQWLPADKLANLYQE
Homo         226 LVKEAIDKAGYTEKIVIGMDVAASEFYRDG--KYDLDFKSP-TDPSRYITGDQLGALYQD
P.berghei    234 LLVASIKKAGYENKVKIAMDVAASEFYNIETKTYDLDFKTPNNDKSLVKTGQELVDLYIE

C.elegans    315 FIKEYPVVSIEDAFDQDDWDNWGKFHGATS--IQLVGDDLTVTNPKRIQTAIDKKSCNCL
S.cervisiae  286 LMKRYPIVSIEDPFAEDDWEAWSHFFKTAG--IQIVADDLTVTNPKRIATAIEKKAADAL
Drosophila   351 FIKDFPIVSIEDPFDQDHWEAWSNLTGCTD--IQIVGDDLTVTNPKRIATAVEKKACNCL
Homo         283 FVRDYPVVSIEDPFDQDDWAAWSKFTANVG--IQIVGDDLTVTNPKRIERAVEEKACNCL
P.berghei    294 LVKKYPIISIEDPFDQDDWENYAKLTEAIGKDVQIVGDDLLVTNPTRIEKALEKKACNAL

C.elegans    373 LLKVNQIGSVTESIEAAKLSRANGWGVMVSHRSGETEDTFIADLVVGLATGQIKTGAPCR
S.cervisiae  344 LLKVNQIGTLSESIKAAQDSFAAGWGVMVSHRSGETEDTFIADLVVGLRTGQIKTGAPAR
Drosophila   409 LLKVNQIGTVTESIAAHLLAKKNGWGTMVSHRSGETEDSFIGDLVVGLSTGQIKTGAPCR
Homo         341 LLKVNQIGSVTEAIQACKLAQENGWGVMVSHRSGETEDTFIADLVVGLCTGQIKTGAPCR
P.berghei    354 LLKVNQIGSITEAIEACLLSQKNNWGVMVSHRSGETEDVFIADLVVALRTGQIKTGAPCR

C.elegans    433 SERLAKYNQLLRIEEELGADAVYAGHNFRNPQV-
S.cervisiae  404 SERLAKLNQLLRIEEELGDNAVFAGENFHHGDKL
Drosophila   469 SERLAKYNQILRIEEEIGAGVKFAGKSFRKPQ--
Homo         401 SERLAKYNQLMRIEEELGDEARFAGHNFRNPSVL
P.berghei    414 SERNAKYNQLFRIEESLGANGSFAGDKFRLQLN-
```
